# The role of captopril in leukotriene deficient type 1 diabetic mice

**DOI:** 10.1038/s41598-023-49449-8

**Published:** 2023-12-13

**Authors:** João Pedro Tôrres Guimarães, Luiz A. D. Queiroz, Kalhara R. Menikdiwela, Nayara Pereira, Theresa Ramalho, Sonia Jancar, Naima Moustaid-Moussa, Joilson O. Martins

**Affiliations:** 1 Laboratory of Immunoendocrinology, School of Pharmaceutical Sciences, Department of Clinical and Toxicological Analyses, University of São Paulo, São Paulo, SP Brazil; 2 Laboratory of Nutrigenomics, Inflammation and Obesity Research, Department of Nutritional Sciences, and Obesity Research Institute, Texas Tech University (TTU), Lubbock, TX USA; 3 Laboratory of Immunopharmacology, Department of Immunology, Institute of Biomedical Sciences, University of São Paulo (ICB/USP), São Paulo, SP Brazil; 4 Present Address: Department of Nutritional Sciences, Rutgers University, New Brunswick, NJ USA; 5 Present Address: Department of Pharmacology, Ribeirão Preto Medical School (FMRP/USP), Ribeirão Preto, SP Brazil; 6 Present Address: Department of Molecular Cell and Cancer Biology, University of Massachusetts Chan Medical School, Worcester, MA USA

**Keywords:** Immunology, Endocrinology

## Abstract

T1D can be associated with metabolic disorders and several impaired pathways, including insulin signaling, and development of insulin resistance through the renin-angiotensin system (RAS). The main precursor of RAS is angiotensinogen (Agt) and this system is often linked to autophagy dysregulation. Dysregulated autophagy has been described in T1D and linked to impairments in both glucose metabolism, and leukotrienes (LTs) production. Here, we have investigated the role of RAS and LTs in both muscle and liver from T1D mice, and its effects on insulin and autophagy pathways. We have chemically induced T1D in 129sve and 129sve 5LO^−/−^ mice (lacking LTs) with streptozotocin (STZ). To further inhibit ACE activity, mice were treated with captopril (Cap). In muscle of T1D mice, treatment with Cap increased the expression of RAS (angiotensinogen and angiotensin II receptor), insulin signaling, and autophagy markers, regardless of the genotype. In the liver of T1D mice, the treatment with Cap increased the expression of RAS and insulin signaling markers, mostly when LTs were absent. 5LO^−/−^ T1D mice showed increased insulin sensitivity, and decreased NEFA, after the Cap treatment. Cap treatment impacted both insulin signaling and autophagy pathways at the mRNA levels in muscle and liver, indicating the potential role of ACE inhibition on insulin sensitivity and autophagy in T1D.

## Introduction

Type 1 diabetes (T1D) is a disease caused by the autoimmune destruction of pancreatic beta cells, which are responsible for producing insulin^1^. In the absence of insulin, a hyperglycemic condition stands, often associated with a low-grade inflammatory profile, however, the impairment in the glucose metabolism can affect not only insulin signaling pathway, but also other important pathways responsible for metabolic and cell homeostasis, such as autophagy^1–3^.

As a result of the chronic low-grade inflammation, some inflammatory mediators may affect the insulin signaling pathway. Leukotrienes (LTs) are lipidic mediators produced by the stimulus of phospholipase A_2_ over the membrane phospholipids, releasing arachidonic acid in the cytoplasm, which will be further converted in leukotriene through the action of the 5-lipoxygenase enzyme (5-LO)^4–6^. LTs are often associated with the inflammatory response; however, some studies have shown that this lipidic mediator can impair the insulin signaling pathway activation, in some metabolic active tissues, such as muscle, liver, and adipose tissue, through the binding of leukotriene B4 (LTB4) in its specific receptor BLT1^7,8^.

LTs are often linked with the development of cardiovascular diseases (CVDs)^9–11^, and T1D patients have increased chances to develop CVDs, which is one of the main mortality-associated causes in this disease^12,13^. The renin-angiotensin system (RAS) is the main physiological system linked with inflammation and CVDs. The primary function of RAS is to regulate blood pressure, yet, the over activation of its components specifically through the action of angiotensinogen II (Ang II) after binding on its angiotensin II type 1 receptor (AT1), contributes to the onset of the inflammatory profile^14–16^. One approach to regulate RAS components and consequently reduce the risk of CVDs is by using angiotensin-converting enzyme (ACE) inhibitors, such as captopril (Cap), known by its direct effects over the ACE enzyme, preventing the production of Ang II and its further proinflammatory actions^17–20^.

Autophagy is a cellular process that is involved with the cell homeostasis and intracellular components cycling, contributing to the cell metabolism^21–23^. Impaired autophagy is linked with the insulin signaling as well as with some diseases, like CVDs and diabetes^3,23,24^. There is a link between the RAS and autophagy process, regarding the activation of their components and further impaired metabolism^25,26^, however there is a gap in the literature in the role of RAS and autophagy pathway in some metabolic active tissues, like muscle and liver, for example, especially their relationship with T1D.

Several studies have analyzed impaired cell metabolism/pathways, especially in the metabolic active tissues, including muscle, liver and adipose tissues, and the role of LTs, but mainly in type 2 diabetes (T2D)^27–31^. We hypothesized that LTs are one of the main factors involved with the impaired autophagy and insulin signaling in muscle and liver during T1D, and that Cap treatment can help to restore the well-functioning of these pathways. Therefore, studying and comparing the main findings in a T1D model could fill the gap between these well studied diseases, since besides its ontology, both T1D and T2D shares important characteristics and pathways that are linked to the progression of the disease. A better understanding of the similarities and differences among them can help to improve not only the health of patients under constant treatment, but also contributes to new treatment findings and approaches.

## Results

### Metabolic profile of 129sve and 129sve 5LO^−/−^ mice treated or not with captopril

**Figure 1:**
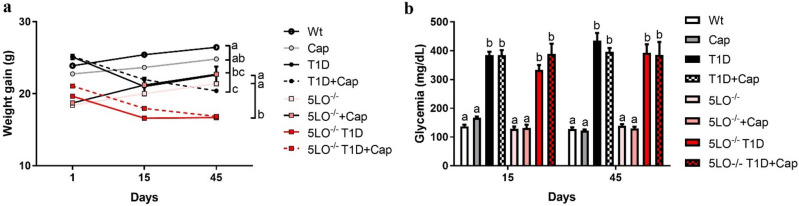
Weight variation and glycemia from 129 and 129sve 5LO^−/−^ mice treated or not with captopril. (**A**) Body weight of Wt, Cap, T1D, T1D + Cap, 5LO^−/−^, 5LO^−/−^ + Cap, 5LO^−/−^ T1D and 5LO^−/−^ T1D + Cap mice groups. (**B**) Blood glucose measurement of Wt, Cap, T1D, T1D + Cap, 5LO^−/−^, 5LO^−/−^ + Cap, 5LO^−/−^ T1D and 5LO^−/−^ T1D + Cap mice groups. The body weight of the mice was evaluated throughout the T1D induction protocol and treatment with Cap. Blood glucose was checked 10 days after the last dose of STZ to confirm the T1D induction and 4 weeks after Cap treatment. Mice that presented weight loss together with blood glucose over 300 mg/dL were considered diabetic. n = 5–7 animals per group. Data are displayed as mean ± SEM. Different superscripts lowercase letters means statistical significance, with* p* being at least < 0.05. One-way ANOVA followed by Bonferroni post-test was used to analyze this set of data.

After verifying STZ-induced hyperglycemia at day 15th, half of the T1D mice groups were treated with Cap daily by gavage (30 mg/L), for 30 days, followed by blood glucose measurements and then also at the 45th day regardless of whether mice are treated with or without Cap. We have considered diabetic mice that presented blood glucose greater than 300 mg/dL. We observed reduced weights in all T1D mice compared to their control groups, regardless of the Cap treatment and the genotype (5LO^−/−^) (Wt vs T1D p = 0.0260; Wt vs T1D + Cap p < 0.0001; 5LO^−/−^ vs 5LO^−/−^ T1D p < 0.0001; 5LO^−/−^ vs 5LO^−/−^ T1D + Cap p < 0.0001) (Fig. 1A). Similarly, T1D mice have remained hyperglycemic, regardless of Cap treatment and genotype (Wt vs T1D p < 0.0001; Wt vs T1D + Cap p < 0.0001; 5LO^−/−^ vs 5LO^−/−^ T1D p < 0.0001; 5LO^−/−^ vs 5LO^−/−^ T1D + Cap p < 0.0001) (Fig. 1B). Mice in the diabetic groups lost weight when compared to their controls and the Cap treatment of 129sve and 129sve 5LO^−/−^ mice did not influence the weight gain of these mice, nor the glycemic control.

### Insulin-related parameters of 129sve and 129sve 5LO^−/−^ mice treated or not with captopril

**Figure 2:**
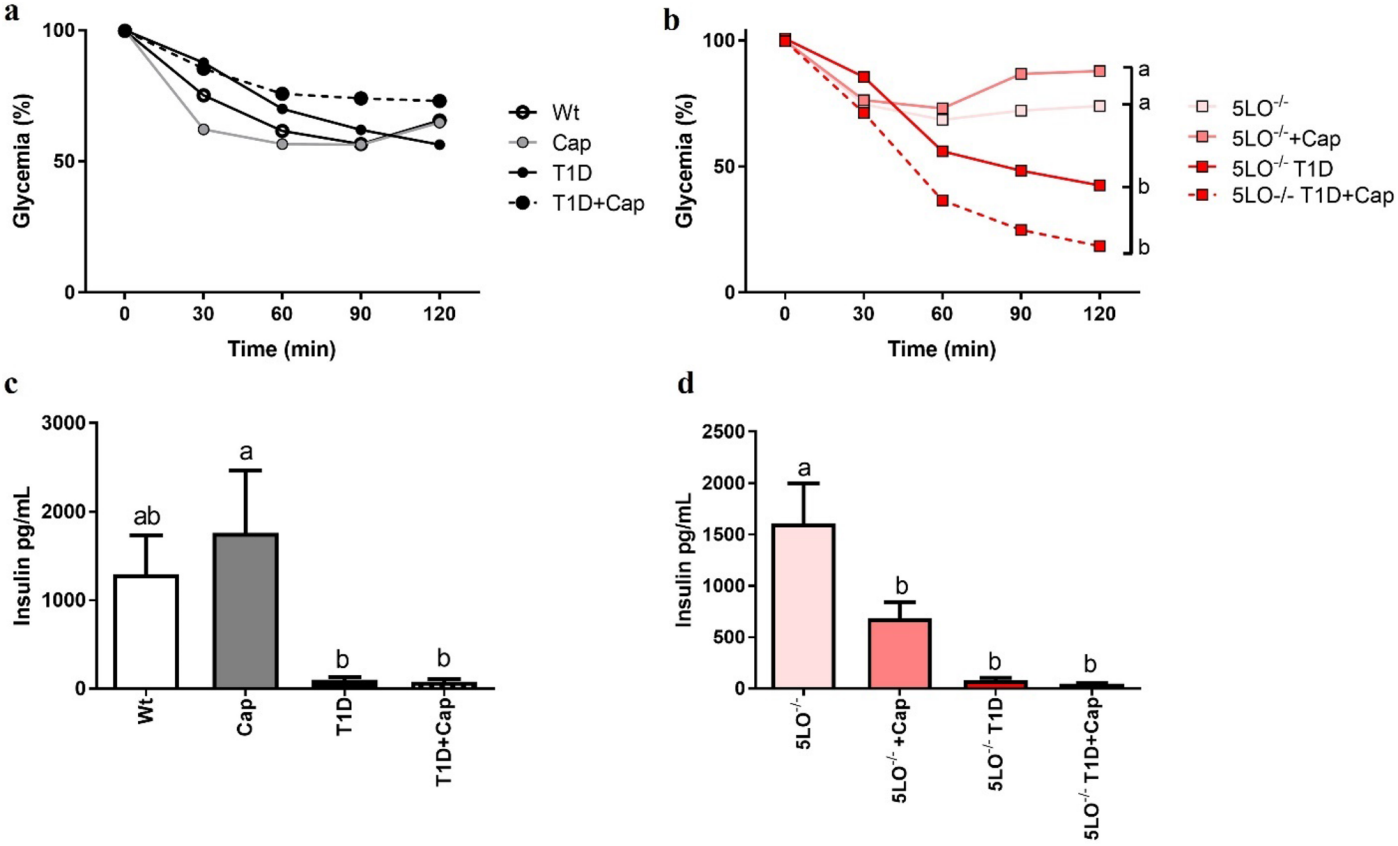
Insulin tolerance test and insulin plasma levels from 129 and 129sve 5LO^−/−^ mice treated or not with captopril. (**A,B**) ITT of Wt, Cap, T1D, T1D + Cap, 5LO^−/−^, 5LO^−/−^ + Cap, 5LO^−/−^ T1D and 5LO^−/−^ T1D + Cap mice groups. C and D: Insulin dosage of Wt, Cap, T1D, T1D + Cap, 5LO^−/−^, 5LO^−/−^ + Cap, 5LO^−/−^ T1D and 5LO^−/−^ T1D + Cap mice groups. After insulin administration (0.75 IU/kg), the percentage of glucose in the blood of mice was measured at 0, 30, 60, 90 and 120 min. Plasma insulin concentration was analyzed after the plasma acquisition from these mice, without any insulin treatment. n = 5–9 animals per group. Data are displayed as means ± SEM. Different superscripts lowercase letters mean statistical significance, with* p* being at least < 0.05. One-way ANOVA followed by Bonferroni post-test was used to analyze this set of data.

Insulin tolerance test was performed to investigate the potential effects of Cap in restoring the insulin sensitivity in diabetic mice. We have observed that 129sve mice have a reduced ability to control glycemia, even after receiving insulin. However, 129sve 5LO^−/−^ T1D and 129sve 5LO^−/−^ T1D mice treated with Cap, had a lower level of blood glucose, after received insulin (129sve groups 0–120 min for all groups: ns; 129sve 5LO^−/−^ groups: 0 and 30 min: ns among the groups; 60 min: 5LO^−/−^ vs 5LO^−/−^ T1D ns p = 0.6975; 5LO^−/−^ vs 5LO^−/−^ T1D + Cap p = 0.0242; 5LO^−/−^ + Cap vs 5LO^−/−^ T1D ns p = 0.4531; 5LO^−/−^ + Cap vs 5LO^−/−^ T1D + Cap p = 0.0073; 90 min: 5LO^−/−^ vs 5LO^−/−^ T1D ns p = 0.1681; 5LO^−/−^ vs 5LO^−/−^ T1D + Cap p = 0.0002; 5LO^−/−^ + Cap vs 5LO^−/−^ T1D p = 0.0063; 5LO^−/−^ + Cap vs 5LO^−/−^ T1D + Cap p < 0.0001; 120 min: 5LO^−/−^ vs 5LO^−/−^ T1D p = 0.0356; 5LO^−/−^ vs 5LO^−/−^ T1D + Cap p < 0.0001; 5LO^−/−^ + Cap vs 5LO^−/−^ T1D p = 0.0008; 5LO^−/−^ + Cap vs 5LO^−/−^ T1D + Cap p < 0.0001) (Fig. 2A, B). Regarding plasma insulinemia in these animals, we observed that, as expected, the insulin concentration in both groups (treated or not with Cap) of 129sve T1D and 129sve 5LO^−/−^ T1D mice was lower when compared to their respective control or treated groups (Wt vs T1D ns p = 0,1654; Wt vs T1D + Cap ns p = 0.1788; Cap vs T1D p = 0.0216; Cap vs T1D + Cap p = 0.0251; 5LO^−/−^ vs 5LO^−/−^ + Cap p = 0.0071; 5LO^−/−^ vs 5LO^−/−^ T1D p < 0.0001; 5LO^−/−^ vs 5LO^−/−^ T1D + Cap p < 0.0001) (Fig. 2C, D). These results suggest that the absence of leukotrienes in T1D mice is linked to increased insulin sensitivity, when combined with Cap treatment.

### Plasma levels of adipokines from 129 and 129sve 5LO^−/−^ mice treated or not with captopril

**Figure 3:**
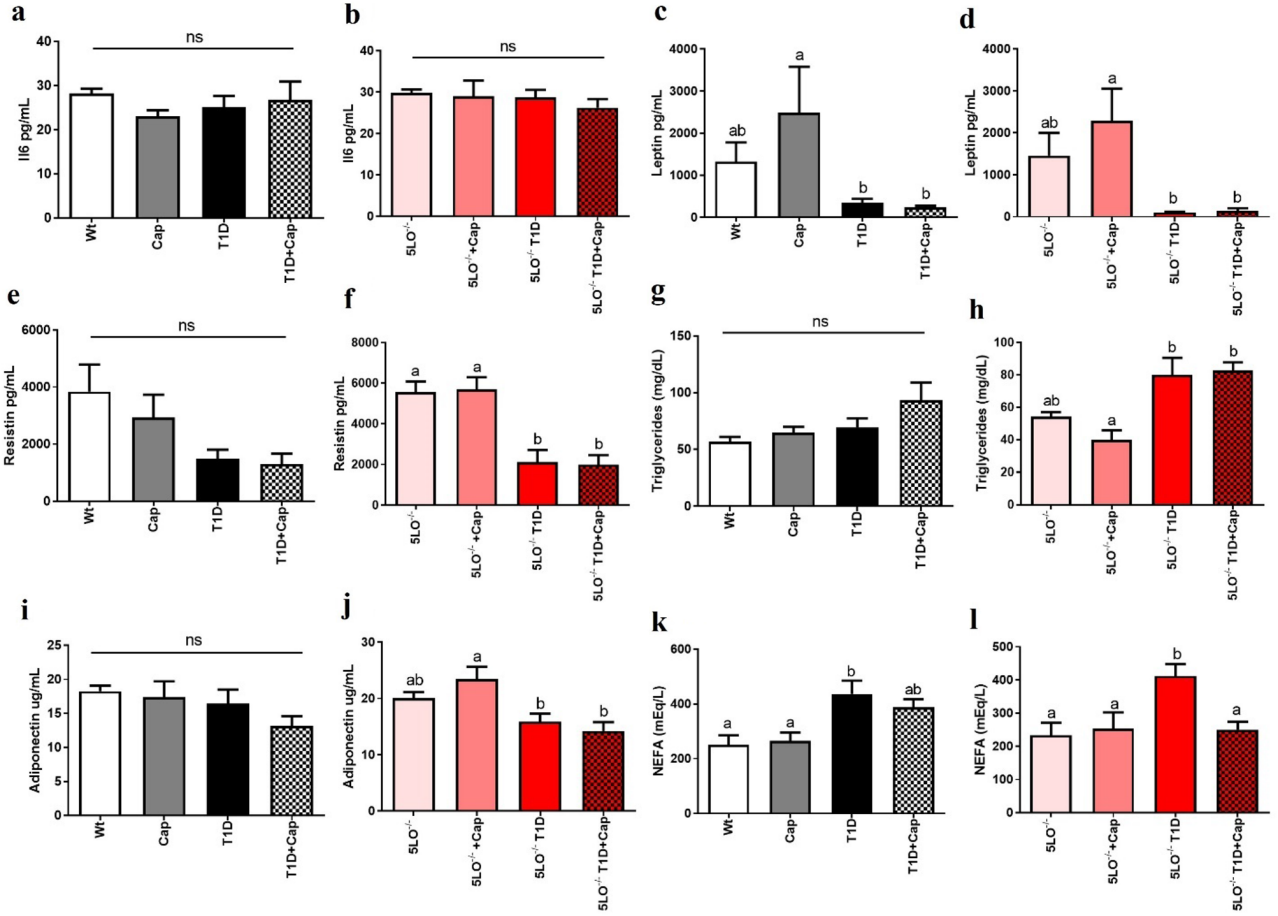
Effects of captopril on adipokines plasma levels from 129 and 129sve 5LO^−/−^ mice. (**A,B**) Plasma concentration of IL-6, (**C,D**) leptin, (**E,F**) resistin, (**G,H**) triglycerides, (**I,J**) adiponectin, (**K,L**) non-steroidal fatty acids (NEFA). The measurement was performed from the plasma of Wt, Cap, T1D, T1D + Cap, 5LO^−/−^, 5LO^−/−^ + Cap, 5LO^−/−^ T1D and 5LO^−/−^ T1D + Cap mice groups. n = 4–9 mice per group. Data are displayed as mean ± SEM. Different superscripts lowercase letters means statistical significance, with* p* being at least < 0.05. One-way ANOVA followed by Bonferroni post-test was used to analyze this set of data.

We did not observe any difference between the groups, regarding the plasma concentration of IL-6, regardless of the treatment with Cap or the genotype (Fig. 3A, B). Interestingly, we observed that leptin plasma concentration was reduced in 129sve T1D (Cap vs T1D p = 0.0458; Cap vs T1D + Cap p = 0.0403) and 129sve 5LO^−/−^ T1D (5LO^−/−^ + Cap vs 5LO^−/−^ T1D p = 0.0010; 5LO^−/−^ + Cap vs 5LO^−/−^ T1D + Cap p = 0.0015) mice, regardless of Cap treatment or genotype (Fig. 3C, D). The plasma concentration of resistin was also reduced, but only in the 129sve 5LO^−/−^ T1D mice regardless of Cap treatment (5LO^−/−^ vs 5LO^−/−^ T1D p = 0.0022; 5LO^−/−^ vs 5LO^−/−^ T1D + Cap p = 0.0019; 5LO^−/−^ + Cap vs 5LO^−/−^ T1D p = 0.0015; 5LO^−/−^ + Cap vs 5LO^−/−^ T1D + Cap p = 0.0012), suggesting that in this case, resistin levels could be affected by LTs (Fig. 3E, F). When evaluating the plasma concentration of triglycerides (TG) in 129sve and 129sve 5LO^−/−^ mice, we have found an increase in TG concentrations only in the plasma of 129 5LO^−/−^ T1D mice regardless of Cap treatment (5LO^−/−^ + Cap vs 5LO^−/−^ T1D p = 0.0151; 5LO^−/−^ + Cap vs 5LO^−/−^ T1D + Cap p = 0.0105) (Fig. 3G, H). Adiponectin plasma levels were decreased on both 129sve 5LO^−/−^ T1D treated or not with Cap (5LO^−/−^ + Cap vs 5LO^−/−^ T1D p = 0.0307; 5LO^−/−^ + Cap vs 5LO^−/−^ T1D + Cap p = 0.0045), but only in this genotype, indicating that LTs absence could be linked to the observed plasma decreased levels of this adipokine (Fig. 3I, J). NEFA plasma concentration was increased only in the plasma of both 129sve T1D (Wt vs T1D p = 0.0240) and 129sve 5LO^−/−^ T1D (5LO^−/−^ vs 5LO^−/−^ T1D p = 0.0217), however, only in the plasma of 129sve 5LO^−/−^ T1D mice treated with Cap, where we were able to observe a decrease in the basal levels of these fatty acids (5LO^−/−^ T1D vs 5LO^−/−^ T1D + Cap p = 0.0284) (Fig. 3K, L). These results suggest that the plasma levels of these adipokines could be affected not only by the presence/absence of LTs, but also by the treatment with Cap.

### Captopril effects over the gene expression of RAS markers in the muscle from 129 and 129sve 5LO^−/−^ mice

**Figure 4:**
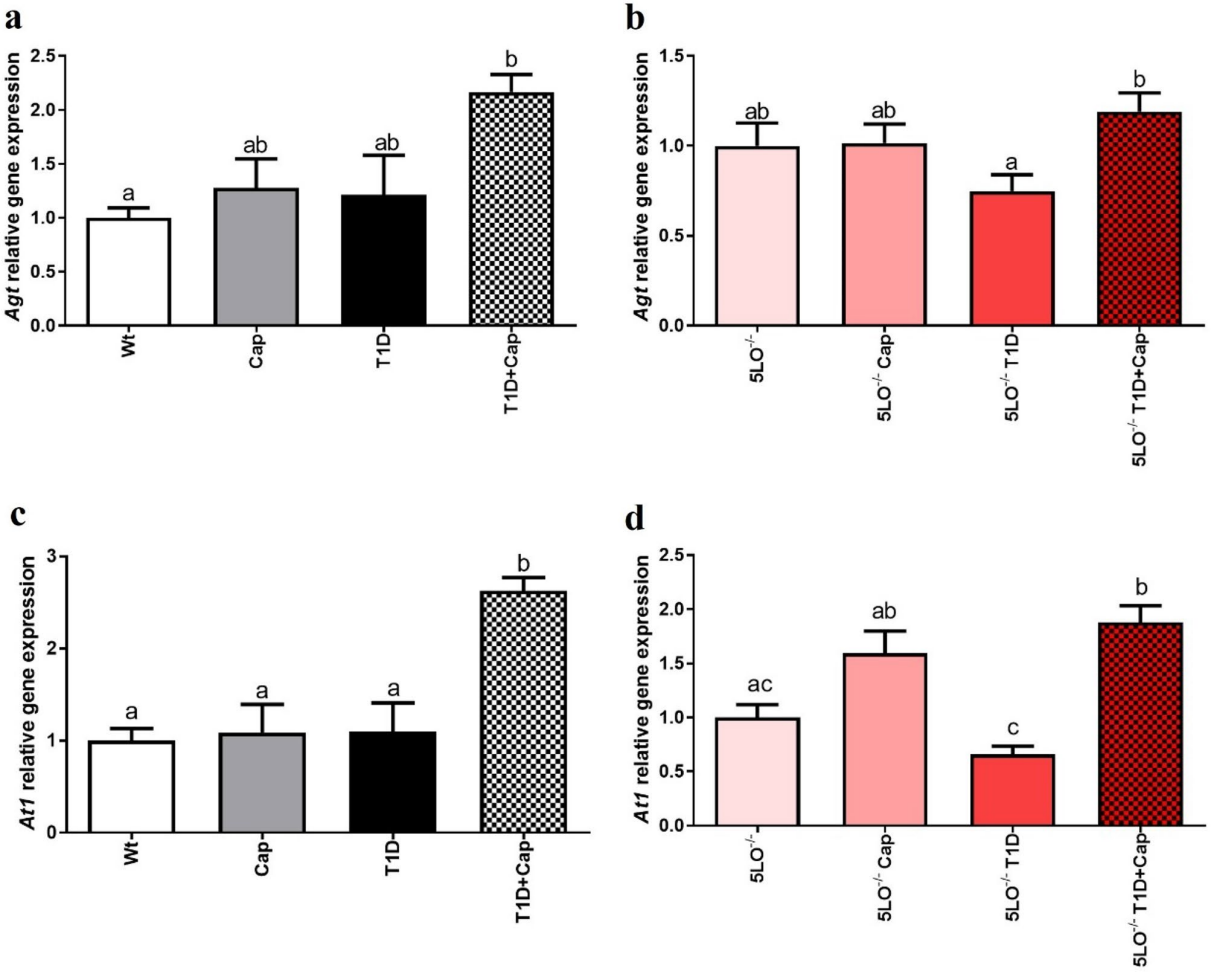
Effects of captopril on the expression of RAS markers in the muscle from 129 and 129sve 5LO^−/−^ mice. (**A,B**) *Agt* gene expression in the muscle of Wt, Cap, T1D, T1D + Cap, 5LO^−/−^, 5LO^−/−^ + Cap, 5LO^−/−^ T1D and 5LO^−/−^ T1D + Cap mice groups. (**C,D**) *At1* gene expression in the muscle of Wt, Cap, T1D, T1D + Cap, 5LO^−/−^, 5LO^−/−^ + Cap, 5LO^−/−^ T1D and 5LO^−/−^ T1D + Cap mice groups. *Agt* and *At1* gene expression was analyzed using muscle homogenate from the groups mentioned. n = 4–7 mice per group. Data are displayed as mean ± SEM. Different superscripts lowercase letters means statistical significance, with* p* being at least < 0.05. One-way ANOVA followed by Bonferroni post-test was used to analyze this set of data.

We have observed increased gene expression of *Agt*, in 129sve T1D (p = 0.0391) and 129sve 5LO^−/−^ T1D mice treated with Cap (p = 0.0484) (Fig. 4A, B). Interestingly, we have also observed a similar pattern in relation to gene expression of *At1* (Wt vs T1D + Cap p = 0.0016; 5LO^−/−^ vs 5LO^−/−^ T1D + Cap p = 0.0064), the gene responsible for encoding the receptor that binds to angiotensin II (Fig. 4C, D). These results indicate that Cap influence both *Agt* and *At1* gene expression in T1D mice regardless of the presence of LTs.

### Captopril effects over the gene expression of RAS markers in the liver from 129 and 129sve 5LO^−/−^ mice

**Figure 5:**
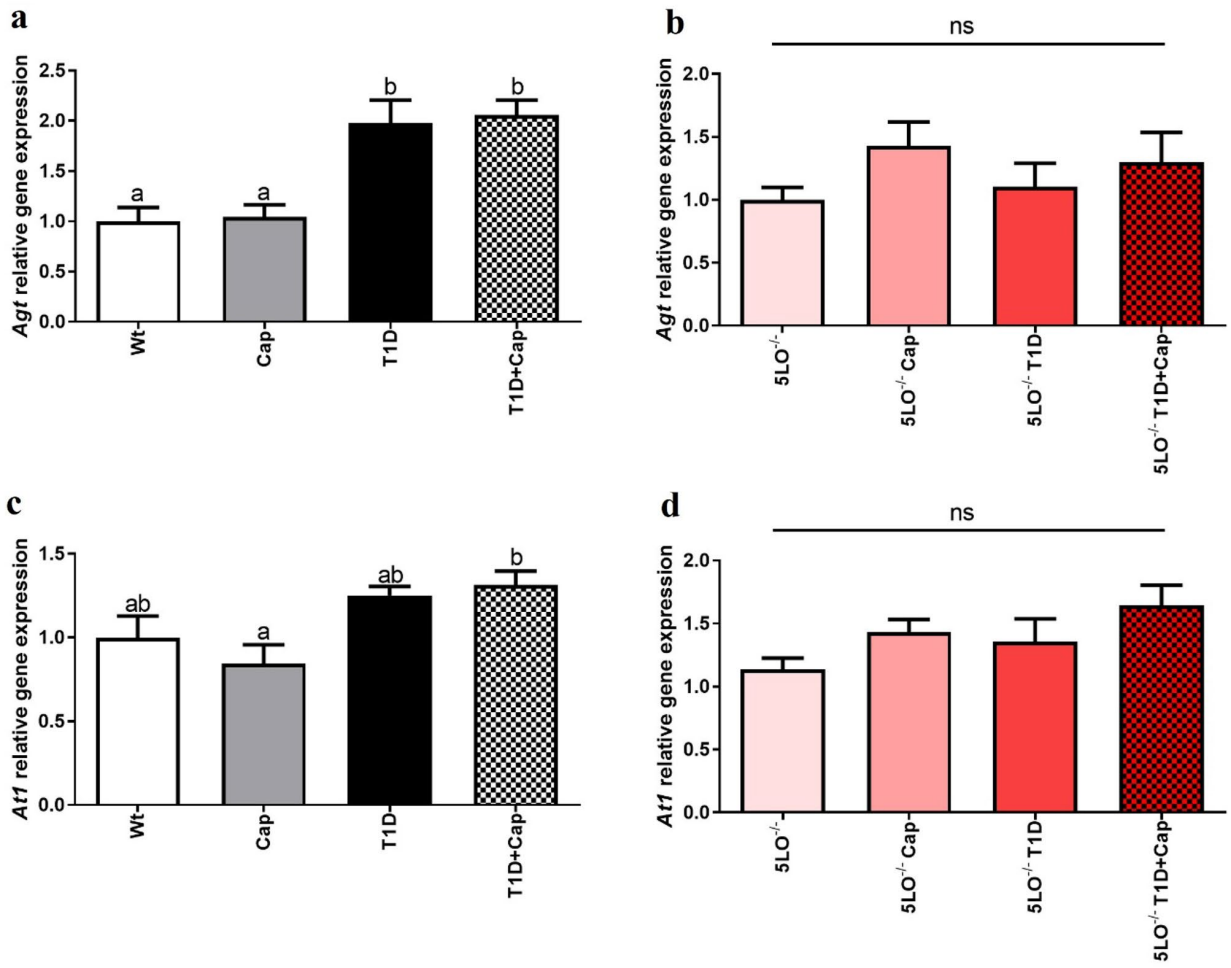
Effects of captopril on the expression of RAS markers in the liver from 129 and 129sve 5LO^−/−^ mice. (**A,B**) *Agt* gene expression in the liver of Wt, Cap, T1D, T1D + Cap, 5LO^−/−^, 5LO^−/−^ + Cap, 5LO^−/−^ T1D and 5LO^−/−^ T1D + Cap mice groups. C and D: *At1* gene expression in the liver of Wt, Cap, T1D, T1D + Cap, 5LO^−/−^, 5LO^−/−^ + Cap, 5LO^−/−^ T1D and 5LO^−/−^ T1D + Cap mice groups. *Agt* and *At1* gene expression was analyzed using liver homogenate from the groups mentioned. n = 5–8 mice per group. Data are displayed as mean ± SEM. Different superscripts lowercase letters means statistical significance, with* p* being at least < 0.05. One-way ANOVA followed by Bonferroni post-test was used to analyze this set of data.

When we evaluated the expression of the main RAS markers in the liver samples, we observed that there was an increase in the expression of *Agt* and *At1*, both in 129sve T1D mice (p = 0.0040) and in 129sve T1D mice treated with Cap (p = 0.0019) for *Agt*, and in 129sve T1D mice treated with Cap (p = 0236) for *At1* and that, differently from what was observed in the muscle, appears to be dependent on the presence of LTs, since there is no differences at gene levels for these markers in the groups from 129 5LO^−/−^ mice (Fig. 5A–D). The Cap treatment on T1D mice, in this case, does not appear to be the cause of the increased expression of these genes, since it was possible to observe that T1D stimulated the expression of *Agt* and *At1* in the liver of these mice.

### Captopril effects over the gene expression of the insulin signaling markers in the muscle from 129 and 129sve 5LO^−/−^ mice

**Figure 6:**
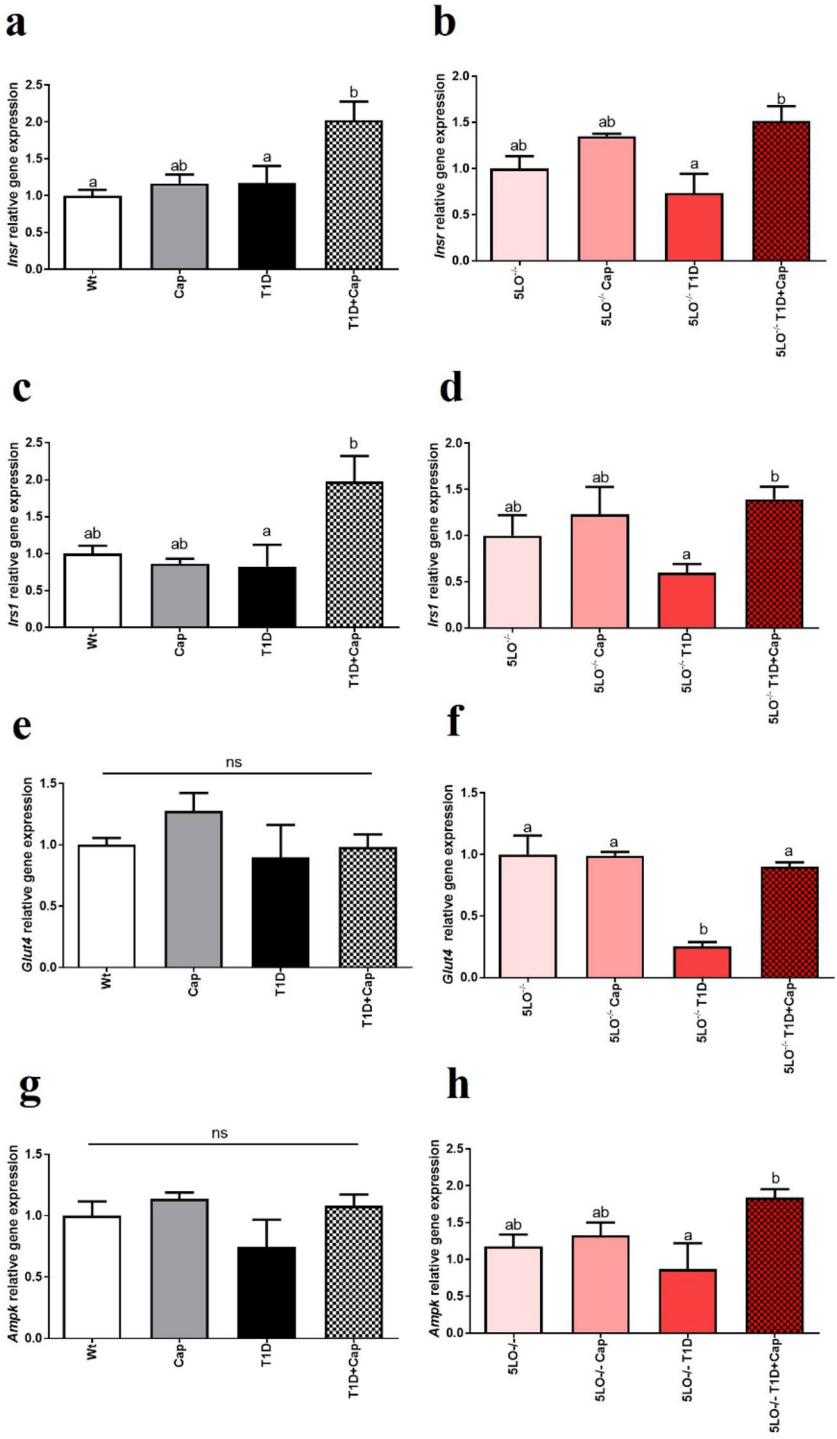
Effects of captopril on the expression of insulin signaling pathway markers in the muscle from 129 and 129sve 5LO^−/−^ mice. (**A,B**) *Insr* gene expression in the muscle of Wt, Cap, T1D, T1D + Cap, 5LO^−/−^, 5LO^−/−^ + Cap, 5LO^−/−^ T1D and 5LO^−/−^ T1D + Cap mice groups. (**C,D**) *Irs1* gene expression in the muscle of Wt, Cap, T1D, T1D + Cap, 5LO^−/−^, 5LO^−/−^ + Cap, 5LO^−/−^ T1D and 5LO^−/−^ T1D + Cap mice groups. (**E,F**) *Glut4* gene expression in the muscle of Wt, Cap, T1D, T1D + Cap, 5LO^−/−^, 5LO^−/−^ + Cap, 5LO^−/−^ T1D and 5LO^−/−^ T1D + Cap mice groups. (**G,H**) *Ampk* gene expression in the muscle of Wt, Cap, T1D, T1D + Cap, 5LO^−/−^, 5LO^−/−^ + Cap, 5LO^−/−^ T1D and 5LO^−/−^ T1D + Cap mice groups. *Insr*, *Irs1*, *Glut4*, and *Ampk* gene expression was analyzed using muscle homogenate from the groups mentioned. n = 4–8 mice per group. Data are displayed as mean ± SEM. Different superscripts lowercase letters means statistical significance, with* p* being at least < 0.05. One-way ANOVA followed by Bonferroni post-test was used to analyze this set of data.

We have noticed that both *Insr* and *Irs1* presented reduced expression in T1D mice muscle, regardless of the genotype, but Cap was able to increase the expression of these genes (*Insr*: Wt vs T1D + Cap p = 0.0106; T1D vs T1D + Cap p = 0.0388; 5LO^−/−^ T1D vs 5LO^−/−^ T1D + Cap p = 0.0137; *Irs1*: T1D vs T1D + Cap p = 0.0376; 5LO^−/−^ T1D vs 5LO^−/−^ T1D + Cap p = 0.0294), in a more accentuated way in muscle from 129sve 5LO^−/−^ mice (Fig. 6A–D). Following a similar pattern, we observed the same phenomenon related both glucose transporter 4 (*Glut4*) and adenosine monophosphate-activated protein kinase (*Ampk*), responsible for glucose uptake, fatty acid oxidation and energy homeostasis in muscles, but only in the muscles of 129sve 5LO^−/−^ mice (*Glut4*: 5LO^−/−^ vs 5LO^−/−^ T1D p < 0.0001; 5LO^−/−^ + Cap vs 5LO^−/−^ T1D p < 0.0001; 5LO^−/−^ T1D vs 5LO^−/−^ T1D + Cap p = 0.0002; *Ampk*: 5LO^−/−^ T1D vs 5LO^−/−^ T1D + Cap p = 0.0219) (Fig. 6E–H). These results suggest that Cap treatment can stimulate and assist in the functioning of the insulin signaling pathway in the muscle of T1D mice, regardless of the LTs presence. We have evaluated the protein expression of both phosphorylated and total AKT and AMPK, however no main changes were observed among the groups in the muscle (Supplementary Fig. 1A–D).

### Captopril effects over the gene expression of the insulin signaling markers in the liver from 129 and 129sve 5LO^−/−^ mice

**Figure 7:**
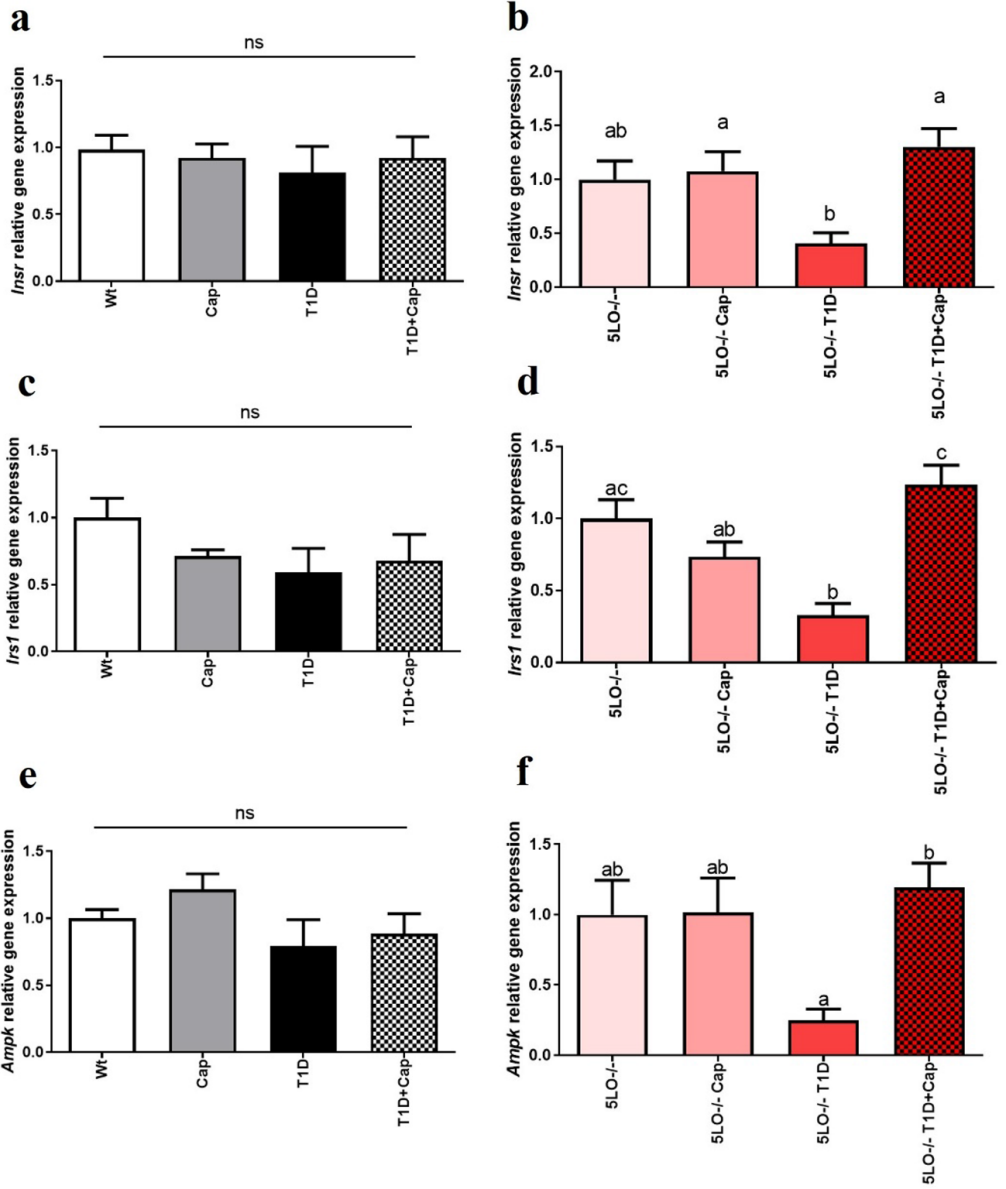
Effects of captopril on the expression of insulin signaling pathway markers in the liver from 129 and 129sve 5LO^−/−^ mice. (**A,B**) *Insr* gene expression in the liver of Wt, Cap, T1D, T1D + Cap, 5LO^−/−^, 5LO^−/−^ + Cap, 5LO^−/−^ T1D and 5LO^−/−^ T1D + Cap mice groups. (**C,D**) *Irs1* gene expression in the liver of Wt, Cap, T1D, T1D + Cap, 5LO^−/−^, 5LO^−/−^ + Cap, 5LO^−/−^ T1D and 5LO^−/−^ T1D + Cap mice groups. (**E,F**) *Ampk* gene expression in the liver of Wt, Cap, T1D, T1D + Cap, 5LO^−/−^, 5LO^−/−^ + Cap, 5LO^−/−^ T1D and 5LO^−/−^ T1D + Cap mice groups. *Insr*, *Irs1*, and *Ampk* gene expression was analyzed using liver homogenate from the groups mentioned. n = 4–7 mice per group. Data are displayed as mean ± SEM. Different superscripts lowercase letters means statistical significance, with* p* being at least < 0.05. One-way ANOVA followed by Bonferroni post-test was used to analyze this set of data.

The expression insulin receptor (*Insr* and *Irs1*) and *Ampk* markers did not show differences among groups in129sve mice liver. However, in the liver of the 129sve 5LO^−/−^ mice, we were able to identify a decrease for these genes expression in the T1D group, but Cap treatment was capable to restore the expression of these genes (*Insr*: 5LO^−/−^ + Cap vs 5LO^−/−^ T1D p = 0.0448; 5LO^−/−^ T1D vs 5LO^−/−^ T1D + Cap p = 0.0029; *Irs1*: 5LO^−/−^ vs 5LO^−/−^ T1D p = 0.0027; 5LO^−/−^ + Cap vs 5LO^−/−^ T1D + Cap p = 0.0396; 5LO^−/−^ T1D vs 5LO^−/−^ T1D + Cap p = 0.0001; *Ampk*: 5LO^−/−^ T1D vs 5LO^−/−^ T1D + Cap p = 0.0128) in the same way that we observed in the muscle of these mice (Fig. 7A–F). These results suggest that Cap treatment can stimulate and assist in the functioning of the insulin signaling pathway, in the same way that was observed with muscle samples, in mice with T1D, and the absence of LTs may be related to the functioning of this pathway on liver. We have evaluated the protein expression of both phosphorylated and total AKT and AMPK, however no main changes were observed among the groups in the liver (Supplementary Fig. 2A–D).

### Captopril effects over the gene expression of the autophagy markers in the muscle from 129 and 129sve 5LO^−/−^ mice

**Figure 8:**
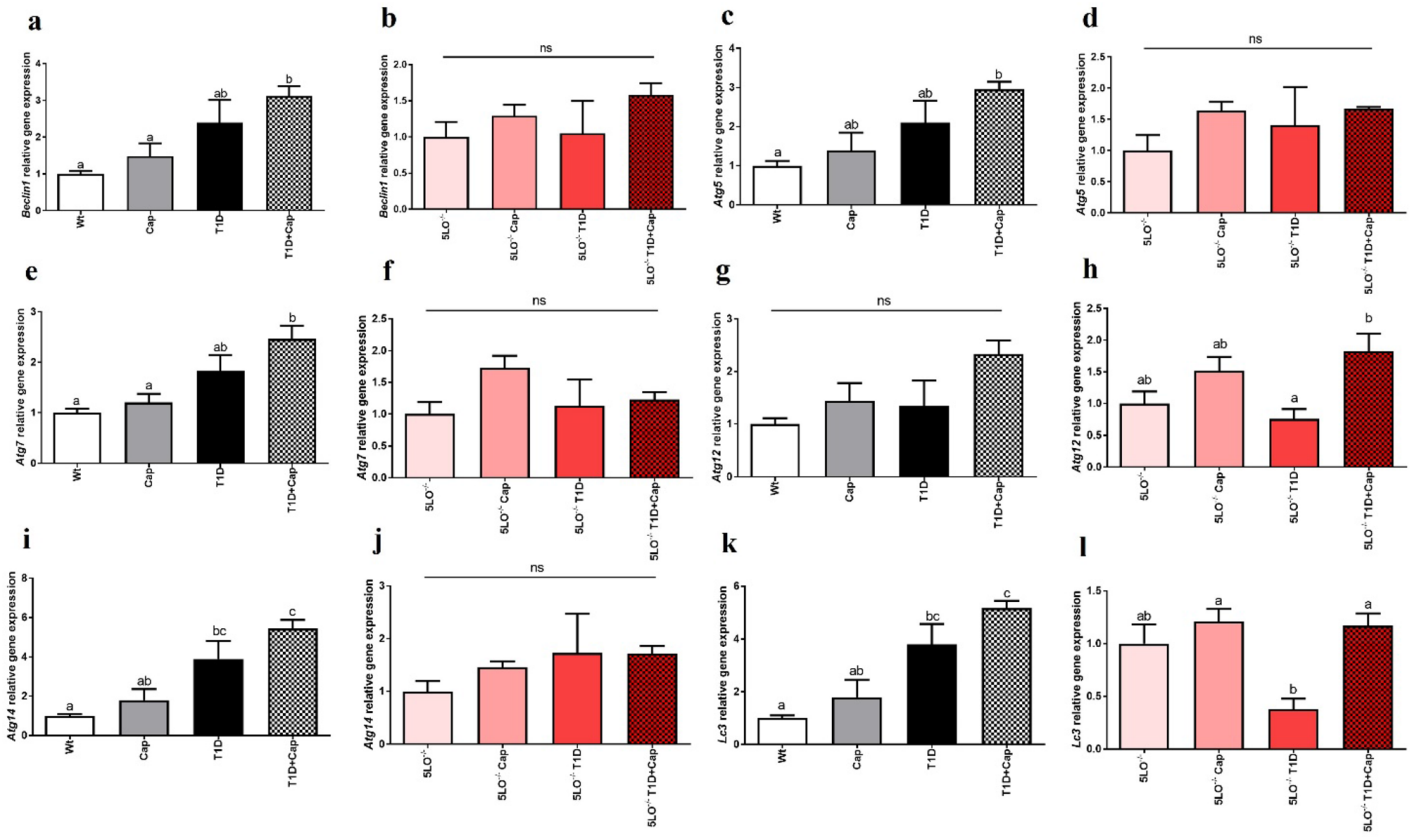
Effects of captopril on the expression of autophagy markers in the muscle from 129 and 129sve 5LO^−/−^ mice. (**A,B**) *Beclin1* gene expression in the muscle of Wt, Cap, T1D, T1D + Cap, 5LO^−/−^, 5LO^−/−^ + Cap, 5LO^−/−^ T1D and 5LO^−/−^ T1D + Cap mice groups. (**C,D**) *Atg5* gene expression in the muscle of Wt, Cap, T1D, T1D + Cap, 5LO^−/−^, 5LO^−/−^ + Cap, 5LO^−/−^ T1D and 5LO^−/−^ T1D + Cap mice groups. (**E,F**) *Atg7* gene expression in the muscle of Wt, Cap, T1D, T1D + Cap, 5LO^−/−^, 5LO^−/−^ + Cap, 5LO^−/−^ T1D and 5LO^−/−^ T1D + Cap mice groups. (**G,H**) *Atg12* gene expression in the muscle of Wt, Cap, T1D, T1D + Cap, 5LO^−/−^, 5LO^−/−^ + Cap, 5LO^−/−^ T1D and 5LO^−/−^ T1D + Cap mice groups. (**I,J**) *Atg14* gene expression in the muscle of Wt, Cap, T1D, T1D + Cap, 5LO^−/−^, 5LO^−/−^ + Cap, 5LO^−/−^ T1D and 5LO^−/−^ T1D + Cap mice groups. (**K,L**) *Lc3* gene expression in the muscle of Wt, Cap, T1D, T1D + Cap, 5LO^−/−^, 5LO^−/−^ + Cap, 5LO^−/−^ T1D and 5LO^−/−^ T1D + Cap mice groups. *Beclin1*, *Atg5*, *Atg7*, *Atg12*, *Atg14*, and *Lc3* gene expression was analyzed using muscle homogenate from the groups mentioned. n = 4–6 mice per group. Data are displayed as mean ± SEM. Different superscripts lowercase letters means statistical significance, with* p* being at least < 0.05. One-way ANOVA followed by Bonferroni post-test was used to analyze this set of data.

Regarding the main markers for the autophagy pathway, we have observed increased expression of some of these markers, where *Beclin1* (p = 0.0029), *Atg5* (p = 0.0101), and *Atg7* (p = 0.0012)from 129sve T1D mice treated with Cap were increased compared to the Wt group, while both 129sve T1D mice showed increased *Atg14* (Wt vs T1D p = 0.0117; Wt vs T1D + Cap p = 0.0001) and *LC3* (Wt vs T1D p = 0.0062; Wt vs T1D + Cap p < 0.0001) expression regardless of Cap treatment, compared to the Wt group (Fig. 8A, C, E, G, I, K). However, in 129 sve 5LO^−/−^ T1D mice, we have only observed a decrease in the expression of *Atg12* (5LO^−/−^ T1D vs 5LO^−/−^ T1D + Cap p = 0.0143) and *LC3* (5LO^−/−^ + Cap vs 5LO^−/−^ T1D p = 0.0130; 5LO^−/−^ T1D vs 5LO^−/−^ T1D + Cap p = 0.0187), and the treatment with Cap restored the expression of these genes (Fig. 8B, D, F, H, J, L), indicating that the presence of LTs may be related to the functioning of this pathway, and that Cap treatment could improve the activation of this pathway. In the same way that we have evaluated some proteins from the previous set of results, we have observed no changes in phosphorylated and total mTOR, or LC3 protein expression in the muscle (Supplementary Fig. 3A–D).

### Captopril effects over the gene expression of the autophagy markers in the liver from 129 and 129sve 5LO^−/−^ mice

**Figure 9:**
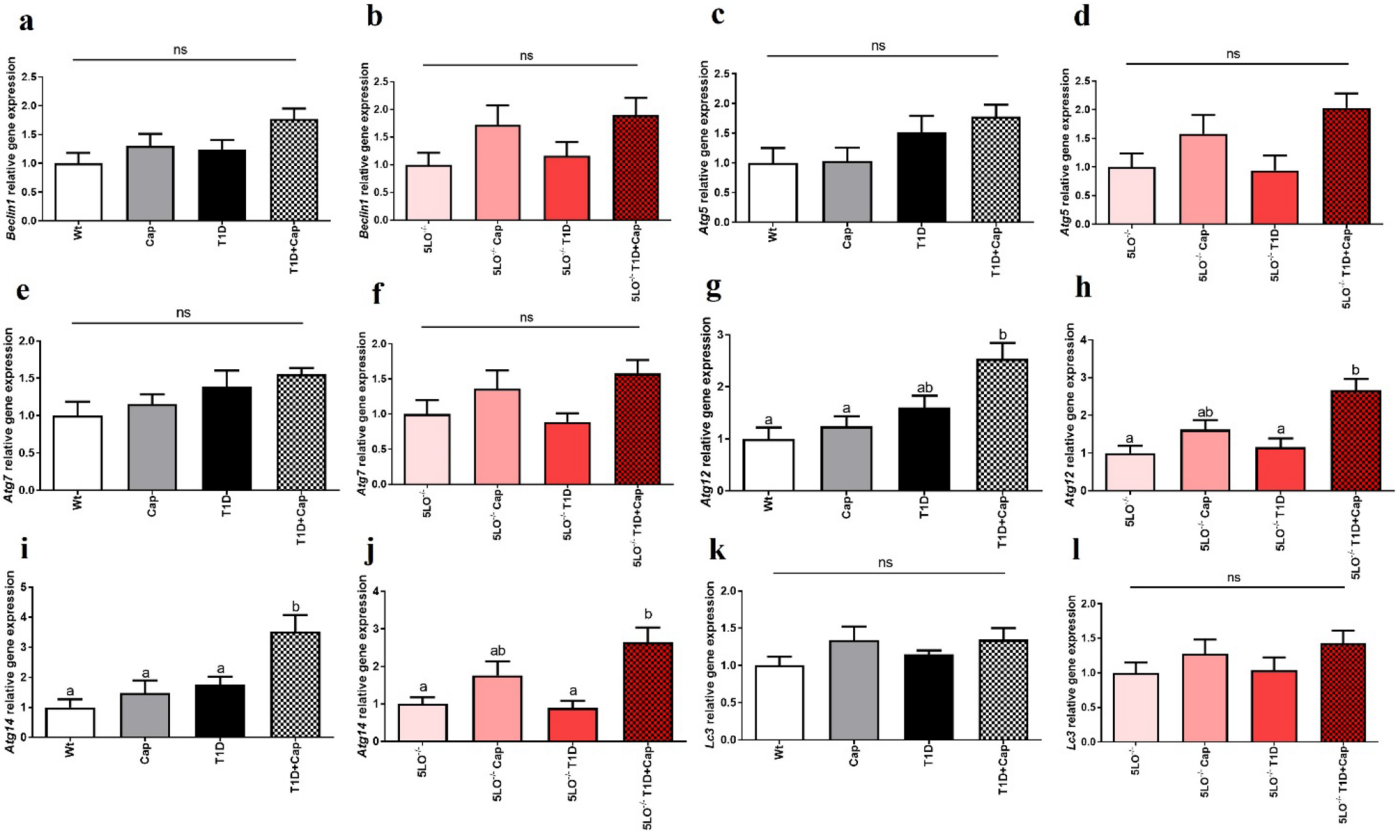
Expression of autophagy markers in the liver from 129 and 129sve 5LO^−/−^ mice treated or not with captopril. (**A,B**) *Beclin1* gene expression in the liver of Wt, Cap, T1D, T1D + Cap, 5LO^−/−^, 5LO^−/−^ + Cap, 5LO^−/−^ T1D and 5LO^−/−^ T1D + Cap mice groups. (**C,D**) *Atg5* gene expression in the liver of Wt, Cap, T1D, T1D + Cap, 5LO^−/−^, 5LO^−/−^ + Cap, 5LO^−/−^ T1D and 5LO^−/−^ T1D + Cap mice groups. (**E,F**) *Atg7* gene expression in the liver of Wt, Cap, T1D, T1D + Cap, 5LO^−/−^, 5LO^−/−^ + Cap, 5LO^−/−^ T1D and 5LO^−/−^ T1D + Cap mice groups. (**G,H**) *Atg12* gene expression in the liver of Wt, Cap, T1D, T1D + Cap, 5LO^−/−^, 5LO^−/−^ + Cap, 5LO^−/−^ T1D and 5LO^−/−^ T1D + Cap mice groups. I and J: *Atg14* gene expression in the liver of Wt, Cap, T1D, T1D + Cap, 5LO^−/−^, 5LO^−/−^ + Cap, 5LO^−/−^ T1D and 5LO^−/−^ T1D + Cap mice groups. K and L: *Lc3* gene expression in the liver of Wt, Cap, T1D, T1D + Cap, 5LO^−/−^, 5LO^−/−^ + Cap, 5LO^−/−^ T1D and 5LO^−/−^ T1D + Cap mice groups. *Beclin1*, *Atg5*, *Atg7*, *Atg12*, *Atg14*, and *Lc3* gene expression was analyzed using liver homogenate from the groups mentioned. n = 4–5 mice per group. Data are displayed as mean ± SEM. Different superscripts lowercase letters means statistical significance, with* p* being at least < 0.05. One-way ANOVA followed by Bonferroni post-test was used to analyze this set of data.

When evaluating the gene expression of some of markers mentioned above in the muscle of these mice, we observed increased *Agt12* (Wt vs T1D + Cap p = 0.0021; Cap vs T1D + Cap p = 0.0087; 5LO^−/−^ vs 5LO^−/−^ T1D + Cap p = 0.0017; 5LO^−/−^ T1D vs 5LO^−/−^ T1D + Cap p = 0.0041) and *Agt14* (Wt vs T1D + Cap p = 0.0019; Cap vs T1D + Cap p = 0.0118; T1D vs T1D + Cap p = 0.0340; 5LO^−/−^ vs 5LO^−/−^ T1D + Cap p = 0.0154; 5LO^−/−^ T1D vs 5LO^−/−^ T1D + Cap p = 0.0134) expression, in both 129sve T1D and 129sve 5LO^−/−^ T1D mice that were treated with Cap, and that, in contrast with the muscle results, occurs independently of the presence of LTs (Fig. 9A–L). Similarly, we have observed no changes in phosphorylated and total mTOR, or LC3 protein expression in the liver from these mice (Supplementary Fig. 4A–D).

## Discussion

Given the high risk for T1D patients to develop cardiovascular diseases, and the established role of impaired insulin and autophagy pathways in these diseases, we proposed to analyze the impact of an ACE inhibitor, Cap on the metabolic impairments in a T1D mouse model. Additionally, LTs stand out as potential mediator impairing insulin and autophagy signaling pathways, both of which that are essential for the cell survival and functions and are known to be impaired in metabolic diseases^6,7,32^. Therefore, we have used a knockout model for LTs (129sve 5LO^−/−^ mice) and their littermates 129sve mice, which we treated with Cap. We evaluated the influence of Cap treatment in presence or absence of LTs on metabolically active organs, namely muscle and liver, from these mice.

During the development of this study, we did find some limitations, for example to address some specific autophagy timepoints, which are important to know the detailed dynamic of this pathway, which can also explain the similar results at protein levels among the groups, but at gene levels we were able to identify some differences, showing that there is an effect of Cap treatment, LTs presence during T1D for autophagy. Also, the results with the phosphorylated proteins involved in the glucose metabolism were not different among the groups, however as one of the main goals of this study was to evaluate the effect of captopril, we decided to not inject the mice before euthanasia with insulin—which is known to triggers AKT phosphorylation, for example. However, these are fields that we are wanting to address in future studies, and the main findings of this current study are very important to lead us to these extra steps that we want to evaluate. Below, we discuss the main findings of this study and the potential link of RAS, insulin and autophagy pathways, along with the role of LTs during T1D and the mentioned pathways.

We have evaluated the insulinemia of wild type vs. STZ-induced diabetic littermates. As expected, it was reduced in the T1D groups, regardless of the presence of LTs or treatment with Cap. However, when we analyzed the ability of 129sve and 129sve 5LO^−/−^ mice to control glycemia after insulin administration, we observed that 129sve T1D mice had difficulty in reducing plasma glucose concentration, even with Cap treatment; however, as previously verified in our work^33^, 129sve 5LO^−/−^ T1D mice were able to better control the glucose levels—and more markedly, in the group that received Cap treatment, indicating the possible role of LTs in reducing functionality of the insulin signaling pathway, as observed in other studies^7,8^, in this case, also in T1D^33^. Furthermore, consistent with our findings, some studies have reported that Cap stimulated the insulin signaling pathway^34–37^, which may improve insulin sensitivity in T1D.

Our results corroborate these findings in the literature not only regarding the response to insulin (ITT) results, but also regarding changes in mRNA markers for the insulin pathway, in both 129sve and 129sve 5LO^−/−^ mice, and the treatment with Cap rescued the expression of these genes, in muscle overall, and more markedly in the absence of LTs, in the liver. The activation of the insulin signaling is mediated by the phosphorylation of the Irs1, which is also the target of pro-inflammatory cytokines and lipid mediators (that are capable to block the serine 473 phosphorylation)^7,8,38^. When LTs binds to its leukotriene B4 (LTB4) receptor 1 (BLT1), it triggers the blockade of the serine 473 phosphorylation of Irs1, which will impair the subsequent phosphorylation of AKT through the activation of PI3K and PDK1—this will block the GLUT translocation to the cell surface, decreasing the cells glucose uptake^7,8^. A similar effect is observed with the binding of the proinflammatory cytokines IL6 and TNFα in their respective receptors^7,8^. Therefore, Cap treatment can stimulate and assist in the role of insulin in metabolic tissues of T1D mice even in the presence of LTs, as captopril treatment help to decrease some pro inflammatory components, which in turn, will no longer block the Irs1 phosphorylation^39–41^.

We did not identify any alterations at the protein expression levels of AKT and AMPK, proteins that are indicative of the functioning of this pathway; however, it is worth mentioning that these animals did not receive any type of stimulus, such as insulin, for example, that would stimulate these signaling pathways and phosphorylation of these molecules^33^, which may explain the absence of alteration in the phosphorylation of these proteins. It is also worth to mention that we intentionally did not add insulin treatment in this work, alongside with Cap treatment, so that we can observe the main effects caused only by the Cap. However, future studies combining Cap and insulin treatments are warranted.

In addition to LTs, other lipid mediators, adipokines and/or hormones important for establishing the pathophysiology of T1D were evaluated. Therefore, we have evaluated the production of the main adipokines in the plasma of our mice groups. Studies have identified that patients with T1D may have high concentrations of TGs and NEFA, among other adipokines, which can be harmful to the pathophysiology of T1D^42–46^. Interestingly, we observed that 129sve 5LO^−/−^ T1D mice had increased concentrations of both TGs and NEFA, and the treatment with Cap contributed to the reduction of NEFA only in the plasma of these mice. Leptin and resistin are hormones related to appetite control^47–49^. An interesting finding was that T1D mice, regardless of Cap treatment, had reduced plasma levels of these hormones. In T1D, weight loss is a common feature; related to this, we observed that our T1D mice had a reduced amount of adipose tissue, which may explain the decreased plasma levels of these two hormones, which are produced by this tissue^50,51^. It is also noteworthy that one common side effect of T1D is polyphagia, due to the lack of keep and process the nutrients from the diet in a proper manner, which is linked with the impaired role of insulin and also due to the action of leptin and resistin, involved in appetite regulation^52,53^.

Decreased plasma levels of adiponectin in 129sve 5LO^−/−^ T1D mice were observed regardless of Cap treatment. Lower concentrations of this adipokine may be related to insulin resistance^54,55^. Treatment with Cap in 129sve 5LO^−/−^ T1D mice was not sufficient to recover the plasma levels of this adipokine. However, further studies are necessary, given the synergy between adiponectin, leptin, and adipokines, to influence the response to insulin; thus, these hormones should not be analyzed individually. Another hypothesis that may explain these results is that both adiponectin and leptinare produced by adipose tissue^56^. Therefore, we must consider the fact that T1D mice lost significant body weight and body fat, which was more notable in the 129sve 5LO^−/−^ mice, and which may explain the low levels of above adipokines.

There is a potential link between RAS and LTs, as during inflammatory responses, not only pro inflammatory cytokines are produced and released, but also some lipidic mediators, such as LTs. This is a positive feedback, as during an inflammatory response, which can have the participation and action of some RAS components, triggers immune and local cells to produce IL6, and TNFα for example, which lead to the action of cells membrane phospholipases that cleaves arachidonic acid, that will be affected by the action of lipoxygenases, releasing LTs, which in turn can stimulate the production and release of these pro inflammatory cytokines^57,58^.

Regarding the RAS pathway and its role in T1D metabolic impairments, we observed increased *Agt* expression, in both muscle and liver from T1D mice, and Cap treatment presented a main role over this effect, however, this increased expression seems to be independent of the presence of LTs, since the expression of this gene was similar for both 129sve and 129sve 5LO^−/−^ mice. Interestingly, a similar pattern for *At1* (responsible for encoding the receptor that binds to Ang II) expression was observed in the muscle, regardless of the presence of LTs, however, the same did not apply to the liver of 129sve 5LO^−/−^ mice, indicating a possible role of LTs in the liver regarding *At1* expression.

As Cap inhibits the conversion of Ang I to Ang II, we would expect some decrease in the expression of these genes but blocking ACE may alternatively activate other pathways that lead to the production of Ang II^59,60^. However, the increased gene expression of RAS related markers in the groups treated with Cap can also be explained by a positive feedback mechanism, since the conversion of Agt into Ang II is being blocked by the action of Cap on ACE, not representing exactly the increase of these components at a protein level.

Autophagy is an important process of cellular homeostasis during periods of cellular stress, in addition to assisting in the recycling processes of organelles and other components present in the cytoplasm, that could be harmful for the cell^61^ which, when impaired, is associated with some metabolic changes involving the lipid accumulation, impaired insulin activity and endoplasmic reticulum (ER) stress, among other dysfunctions^23,61^, for example in the liver tissue^62^, whereas in muscle the same pattern is observed in an opposite way, through a activated autophagy process^63,64^, therefore, depending on the tissue, the increase or decrease in autophagic activity can be beneficial or harmful to the tissue. In diabetic patients, increased autophagy markers has been reported^65^ and it is known that when the autophagy process is impaired, it could be also linked with the establishment of insulin resistance, thus participating in the chronicity of the hyperglycemia in those patients^3,63,66^. We observed that, in 129sve 5LO^−/−^ T1D mice, there was a decrease in the expression of *Atg12* and *LC3*, indicating that the presence of LTs can interfere with the function of this pathway.

There is a relationship between RAS products and autophagy through the activation of the AT1 receptor via Ang II. However, the function triggered by this activation depends on the cell type involved, which reinforces the need for more studies understanding the molecular mechanisms involved in these processes^67^. The way in which the RAS and the autophagy process occur has already been demonstrated in studies involving other cell types, mainly endothelial, neuronal, and cardiac cells, however the relationship between these processes in other metabolically active tissues, such as muscle and liver, still requires investigation to understand the role of RAS and autophagy in these tissues, during the onset of T1D^68–72^.

It is worth mentioning that when autophagy is activated in excess or is reduced, it can lead to complications and therefore, the increased expression observed for some of these genes in the 129sve 5LO^−/−^ T1D mice that received the Cap treatment does not necessarily indicate harmful activity of this pathway. Furthermore, the expression of these genes in the muscle of 129 sve 5LO^−/−^ mice was lower when compared to the levels of 129sve mice. In muscle, in some cases, we observed increased gene expression of autophagy markers in T1D mice, which was different from what we observed in the muscle; moreover, activation of this pathway occurs independently of LTs.

## Conclusions

Our results indicate that ACE inhibition (Captopril treatment) affects the main metabolic signaling pathways in both muscle and liver from T1D mice, and that LTs impair these pathways in this disease. Nevertheless, Cap treatment rescued the expression of markers of the RAS, insulin signaling and autophagy pathways, indicating a potential role of Cap to overcome some of LTs metabolic effects in T1D. A better understanding of interactions among these molecular pathways, and the role of Cap, may lead to developing potential therapeutic targets inT1D.

## Material and methods

### Research design

To address the main hypothesis of our study, we have used a total of 8 mice groups, containing 129sve and 129sve 5LO^−/−^, with or without captopril treatment and with or without T1D. During the onset of diabetes and before we euthanized the mice, we have measured some metabolic parameters, such as blood glucose levels, body weight gain, and insulin tolerance tests, to further confirm not only that the T1D induction was successful, but also to evaluate if the absence of leukotrienes and captopril intervention would help to maintain these parameters or ameliorate some of these characteristics (decreased body weight, increased glucose levels and insulin tolerance). After the euthanasia, we have collected plasma, muscle, and liver to evaluate the effects of leukotrienes and captopril in the plasma insulin levels, followed by some adipokines that are important to the metabolic profile of the mice, and are generally altered during T1D, plus being linked to cardiopathies—a side effect of T1D, and also a common target of ACE inhibitors treatment^73–76^. Then we have evaluated at both gene and protein levels the expression of the main markers of renin-angiotensin system, insulin and autophagy signaling pathways, as we have previously observed that they crosstalk and are involved with the metabolic profile of these target tissues^77^, but in this study we would like to evaluate the role of leukotrienes in the regulation of these pathways during T1D. The detailed information of the methodology mentioned in this research design topic is available in the next subsections.

### Mouse studies

8-week-old, male, 129sve and 129sve 5LO^−/−^ mice were used for this study, being housed in a controlled environment according to the Ethical Committee on Animal Use (CEUA) guidelines, with food restriction periods due to the STZ treatment. The T1D induction on mice was performed after a fasting period of 5 h prior to the intraperitoneal (i.p.) injection of STZ (ChemCruz® U-9889, lot F1816) (65 mg/kg diluted in 0.1 M citrate buffer, pH 4.5) during 5 consecutive days; after 1 h of STZ administration, the food availability was restored. To consider the efficacy of our T1D chemical inducing protocol, mice glycemia was measured and mice presenting glycemia greater than 300 mg/dL (OneTouch^®^ Select Simple™) after 10 days from the last STZ dose were considered diabetic. Citrate buffer was administrated in mice from the control groups. The Cap treatment (30 mg/kg diluted in drinking water) was performed daily by gavage for 4 weeks and started after the glycemia measurement on the 15th day. This study was approved and performed in accordance with the guidelines of CEUA—Institute of Biomedical Sciences (ICB) of the University of São Paulo (USP) (CEUA no. CEUA 7465081118), the Brazilian National Council for the Control of Animal Experimentation (CONCEA). Mice samples were further collected at the end of the STZ/Cap protocol and analyzed at plasma, gene, and protein levels at Texas Tech University. We have selected the 5LO−/− T1D mice as our group have previously shown that LTs are important on T1D parameters such as insulin resistance and inflammation^78–80^, but we did not elucidated the role of LTs in other related pathways such as autophagy, as well as the potential benefits of captopril treatment in restoring the evaluated parameters, which are potentially impaired due to the presence of LTs. All procedures were performed in accordance with the ARRIVE guidelines.

### Insulin tolerance test (ITT)

Mice were fasted for 6 h, followed by an i.p. administration of insulin (0.75 IU/kg). After that, the blood glucose was measured (OneTouch^®^ Select Simple™) from the tail vein by an interval of every 30 min, for 2 h.

### Gene expression

Muscle and liver RNA was isolated with RNeasy mini kit (Qiagen, Valencia, CA, USA), followed by the cDNA synthesis by iScript reverse transcription supermix (BioRad, Hercules, CA, USA). Real-time quantitative polymerase chain reaction (RT-qPCR) using SYBR Green Master Mix (BioRad, Hercules, CA, USA) was performed to analyze the gene expression of the main targets from RAS, insulin signaling and autophagy pathways. The expression of target genes was normalized by 18S housekeeping gene. The Supplementary Table 1 shows the primers sequences used for this study.

### Western blotting

For the protein analysis, we have performed the Bradford protein assay (Bio-Rad, Hercules, CA, USA) to quantify the muscle and liver protein concentration. After the total protein quantification, 10% SDS-PAGE was conducted and the proteins on each gel were transferred overnight to a Polyvinylidene Difluoride (PVDF) membrane (Millipore, Burlington, MA, USA). The membranes were incubated on the next day with the primary antibodies with constant shaking at 4 °C overnight. The day after the primary antibody incubation was followed by an anti-rabbit secondary antibody (Jackson Immuno Research Laboratories, West Grove, PA, USA) incubation for 1 h, and the fluorescence detection was captured with the LI-COR Odyssey machine (LI-COR Odyssey CLX, Lincoln, NE, USA). Rabbit anti- phospho-AKT, phospho-AMPK, phospho-mTOR, LC3, total AKT, total AMPK, total mTOR, and TBP (housekeeping) were the main targets chosen for the protein expression experiments (Cell Signaling Technology, Danvers, MA, USA). After revealed the target protein, the membranes were submitted to a stripping treatment to be re stained with the next antibody against the next target protein.

### Triglyceride dosage

Triglyceride Colorimetric Assay Kit (Cayman Chemical, Ann Arbor, MI, USA) was performed according to manufacturer’s guidelines to quantify the plasma concentration of triglycerides from mice plasma samples.

### Non-steroidal fatty acids dosage

Wako NEFA-HR Kit (Wako Pure Chemical Industries, Ltd., Richmond, VA, USA) was performed according to manufacturer’s guidelines to quantify the plasma concentration of non-steroidal fatty acids from mice plasma samples.

### Multiplex assay

Luminex XMAP technology Magpix™, Milliplex^®^ MAP Mouse Metabolic Hormone Expanded Panel (EMD Millipore, Billerica, MA, USA, Cat. # MMHE-44K) was used to quantify the plasma concentration of interleukin 6 (IL6), leptin, total insulin, and resistin, while Luminex XMAP technology Magpix™, Milliplex^®^ MAP Mouse Adipokine Magnetic Bead Single Plex Kit (EMD Millipore, Billerica, MA, USA, Cat. #MADPNMAG-70K01) was used to quantify the adiponectin plasma concentration from mice samples. Multiplex assays were performed according to the manufacturer’s guidelines.

### Statistics

This study has its data analyzed by analysis of variance (ANOVA) followed by Bonferroni post-test using the GraphPad Prism 6.0 software (La Jolla, CA, USA) and two-tailed p values with 95% confidence intervals were acquired. The data were represented as the mean ± standard error. Values of p < 0.05, p < 0.01, and p < 0.001 were considered significant, as marked in all the figures with different superscript letters, where shared letters represent no significance among the groups.

### Supplementary Information

Supplementary Information.

## Data Availability

The data in this study is available upon reasonable request to the corresponding authors: Joilson O. Martins (martinsj@usp.br) and Naima Moustaid-Moussa (naima.moustaid-moussa@ttu.edu). In addition, Data availability repository link: https://figshare.com/s/49bd2a835a6bfba807ab.
