# Supplementary material for: The role of captopril in leukotriene deficient type 1 diabetic mice
Source: Sci Rep. 2023 Dec 13;13:22105. doi: 10.1038/s41598-023-49449-8 (PMC10719306; doi:10.1038/s41598-023-49449-8)

**Supplementary material**

**The Role of Captopril in Leukotriene Deficient Type 1 Diabetic Mice**

João Pedro Tôrres Guimarães^1,2,3^, Luiz A. D. Queiroz^1^, Kalhara R. Menikdiwela^2,+^, Nayara Pereira^3^^, Theresa Ramalho^3#^, Sonia Jancar^3^, Naima Moustaid-Moussa^2^*, Joilson O. Martins^1^*

^1^ Laboratory of Immunoendocrinology, School of Pharmaceutical Sciences, Department of Clinical and Toxicological Analyses, University of São Paulo, São Paulo, SP, Brazil.

^2^ Laboratory of Nutrigenomics, Inflammation and Obesity Research, Department of Nutritional Sciences, and the Obesity Research Institute, Texas Tech University (TTU), Lubbock, TX, USA.

^3^ Laboratory of Immunopharmacology, Department of Immunology, Institute of Biomedical Sciences, University of São Paulo (ICB/USP), São Paulo, SP, Brazil.

^+^Current address: Department of Nutritional Sciences, Rutgers University, New Brunswick, NJ 08901, USA.

^^^Current address: Department of Pharmacology, Ribeirao Preto Medical School (FMRP/USP), Ribeirao Preto, SP, Brazil.

^#^Current address: Department of Molecular Cell and Cancer Biology, University of Massachusetts Chan Medical School, Worcester, MA 01605, USA.

* Corresponding authors (Equal contributions): Joilson O. Martins ([martinsj@usp.br](mailto:martinsj@usp.br)) and Naima Moustaid-Moussa ([naima.moustaid-moussa@ttu.edu](mailto:naima.moustaid-moussa@ttu.edu))


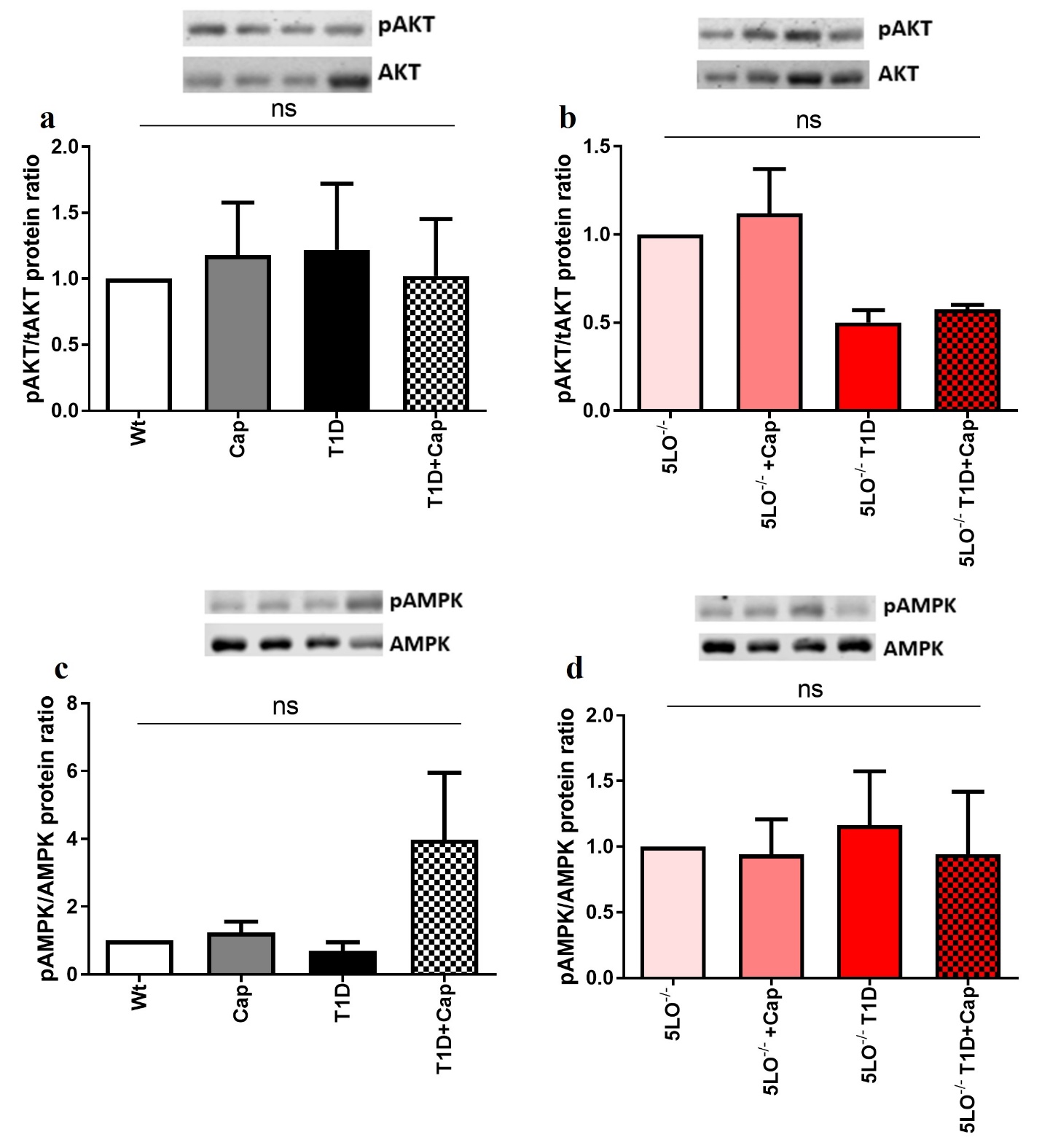
**Supplementary figure 1. Protein expression of markers related to glucose metabolism in muscle from 129sve and 129sve 5LO^-/-^ mice treated or not with captopril.** Protein expression of pAKT and pAmpk were analyzed from muscle (A-D) homogenate after being processed as described in material and methods section 2.4, of Wt, Cap, T1D, T1D+Cap, 5LO^-/-^, 5LO^-/-^+Cap, 5LO^-/-^ T1D and 5LO^-/-^ T1D+Cap mice groups. n= 4-5 mice in each group. One-way ANOVA with Bonferroni post-test.


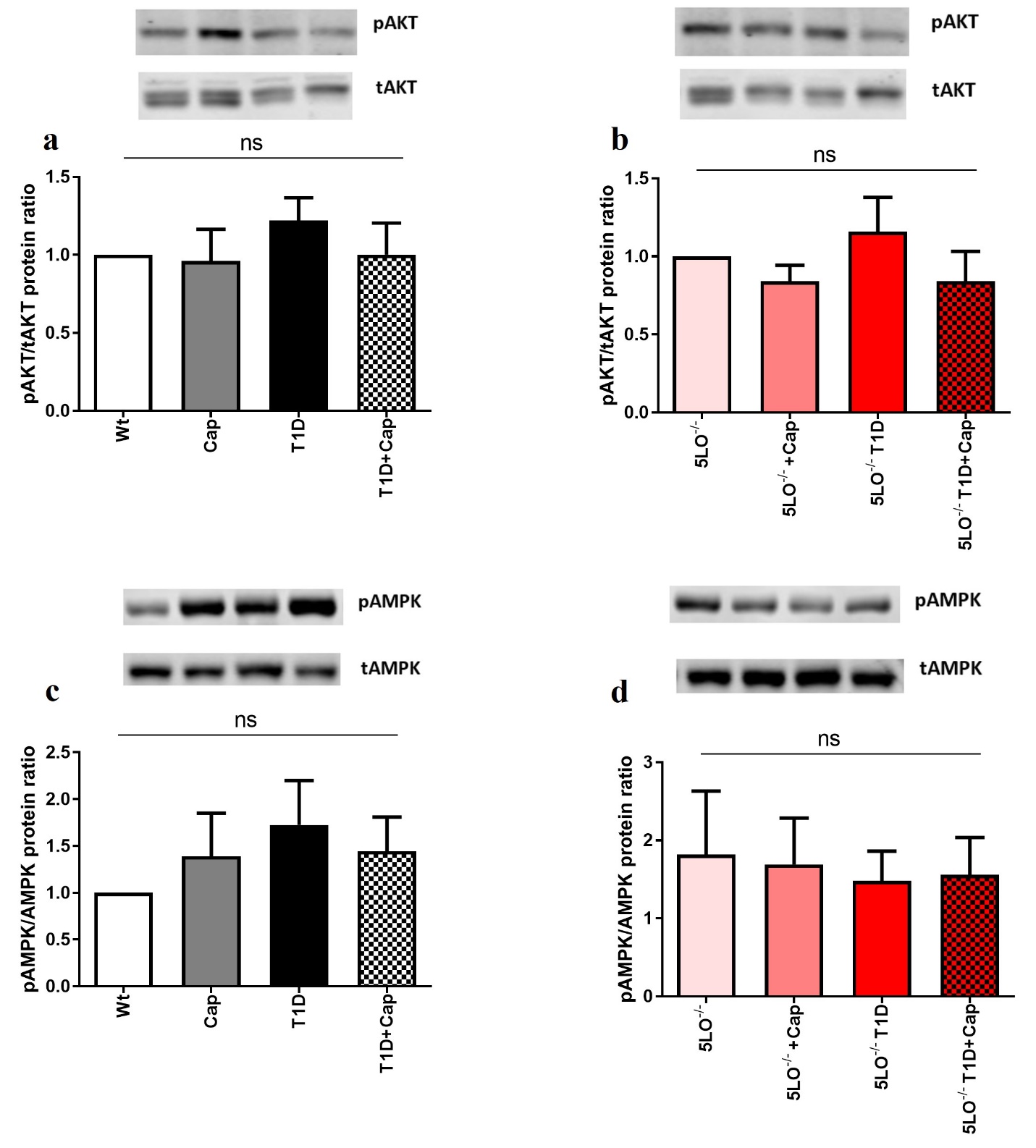
**Supplementary figure 2. Protein expression of markers related to glucose metabolism in liver from 129sve and 129sve 5LO^-/-^ mice treated or not with captopril.** Protein expression of pAKT and pAmpk were analyzed from liver (A-D) homogenate after being processed as described in material and methods section 2.4, of Wt, Cap, T1D, T1D+Cap, 5LO^-/-^, 5LO^-/-^+Cap, 5LO^-/-^ T1D and 5LO^-/-^ T1D+Cap mice groups. n=5 mice in each group. One-way ANOVA with Bonferroni post-test.


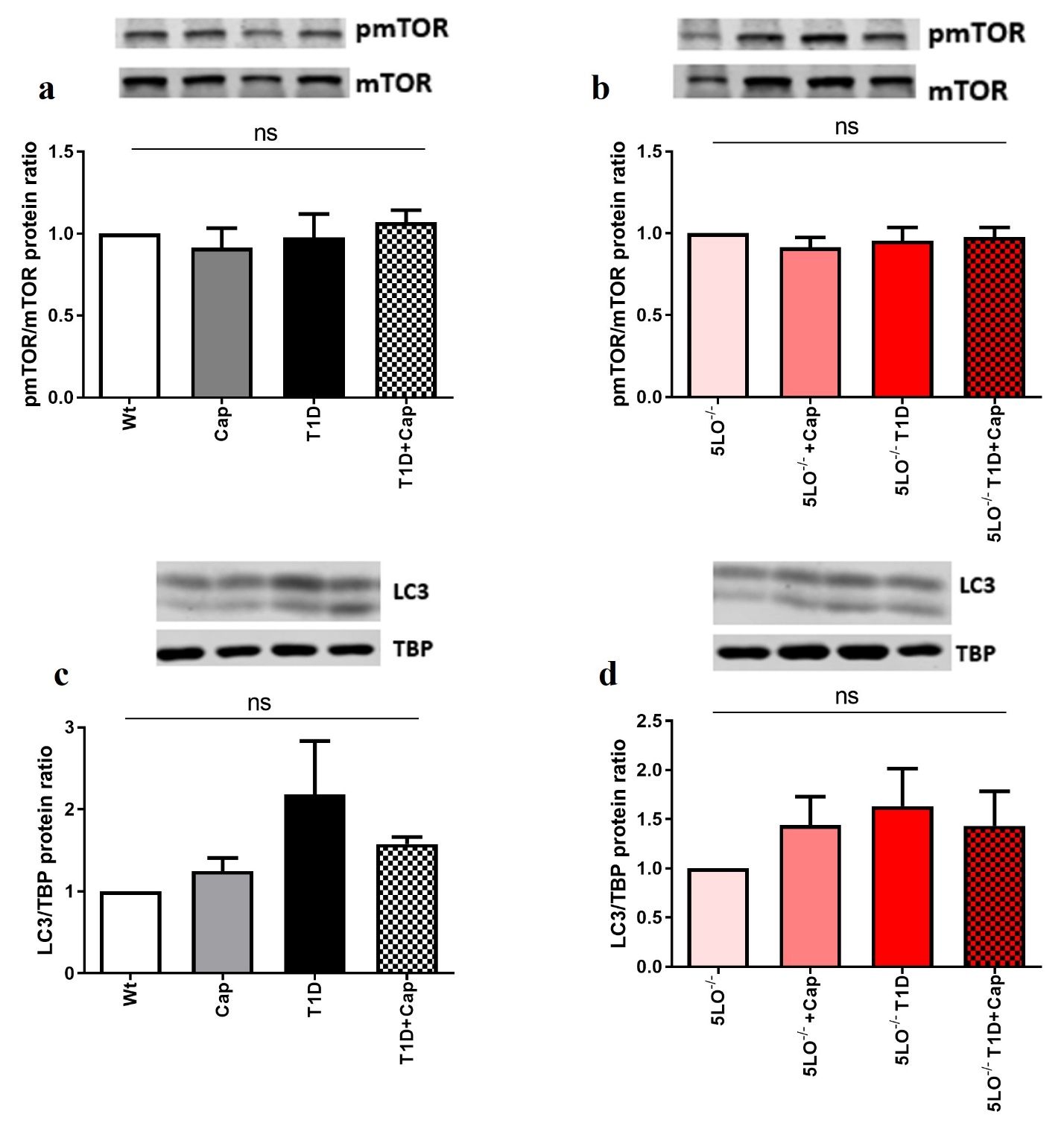
**Supplementary figure 3. Protein expression of markers related to autophagy in muscle from 129sve and 129sve 5LO^-/-^ mice treated or not with captopril.** Protein expression of pmTOR and LC3 were analyzed from muscle (A-D) homogenate after being processed as described in material and methods section 2.4, of Wt, Cap, T1D, T1D+Cap, 5LO^-/-^, 5LO^-/-^+Cap, 5LO^-/-^ T1D and 5LO^-/-^ T1D+Cap mice groups. n= 5 mice in each group. One-way ANOVA with Bonferroni post-test.


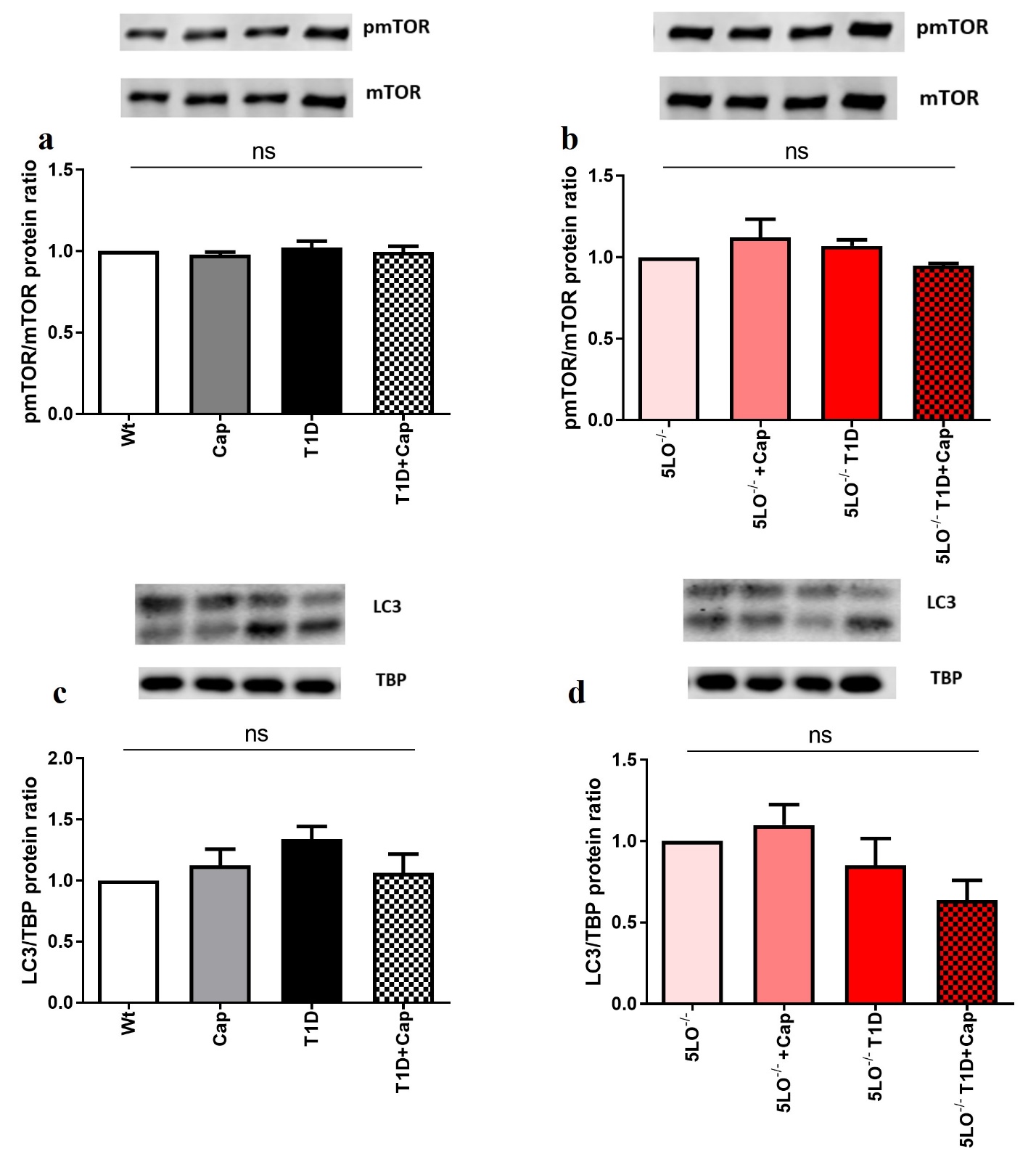
**Supplementary figure 4. Protein expression of markers related to autophagy in liver from 129sve and 129sve 5LO^-/-^ mice treated or not with captopril.** Protein expression of pmTOR and LC3 were analyzed from liver (A-D) homogenate after being processed as described in material and methods section 2.4, of Wt, Cap, T1D, T1D+Cap, 5LO^-/-^, 5LO^-/-^+Cap, 5LO^-/-^ T1D and 5LO^-/-^ T1D+Cap mice groups. n= 5 mice in each group. One-way ANOVA with Bonferroni post-test.

**Supplementary table 1. Primer list of the markers for further gene expression of RAS, glucose metabolism and autophagy.** List of primers and sequences used for the gene expression with muscle and liver samples from Wt, Cap, T1D, T1D+Cap, 5LO^-/-^, 5LO^-/-^+Cap, 5LO^-/-^ T1D and 5LO^-/-^ T1D+Cap mice groups.

| **Primer** | **Sequence** |
| --- | --- |
| ***18S*** | **F: GGACAGGATTGACAGATTGATAGC** |
|  | **R: TGCCAGAGTCTCGTTCGTTA** |
| ***Agt*** | **F: CTGCTGGCTGAGGACAAG** |
|  | **R: CGAGGAGGATGCTATTGAGAAC** |
| ***At1*** | **F: CACTCAAGCCTGTCTACGA** |
|  | **R: TGTCACTCCACCTCAGAAC** |
| ***Insr*** | **F: ACCTTCCAGTATGTTCCTCAG** |
|  | **R: TGCCTTCAGTCATTACCTCTT** |
| ***Irs1*** | **F: TCGCTAACTGAGATAGTCATACAA** |
|  | **R: TCCTGCTAACATCCACCTT** |
| ***Ampk*** | **F: TCAGCACTCCGACAGACTT** |
|  | **R: GGCATCCAGCAGCACATT** |
| ***Glut4*** | **F: AGAGCGTCCAATGTCCTT** |
|  | **R: CGAAGATGCTGGTTGAATAGTAG** |
| ***Beclin1*** | **F: GAGATTGGACCAGGAGGAA** |
|  | **R: AGGTGGCATTGAAGACATTG** |
| ***Atg5*** | **F: TCAGAAGGTTATGAGACAAGAAGA** |
|  | **R: GGATGGACAGTGTAGAAGGT** |
| ***Atg7*** | **F: CCAGGAGATTCAACCAGA** |
|  | **R: GCAGGACAGAGACCATCA** |
| ***Atg12*** | **F: AGCAGGAAGAGTGAACCA** |
|  | **R: AAGCACATAGAGACGAGAAGT** |
| ***Atg14*** | **F: GGCTGGAGTCTGTTCTGT** |
|  | **R: TTGCTGTAGGCGGTAGTT** |
| ***Lc3*** | **F: CTTCTTCCTGCTGGTCAAC** |
|  | **R: TCTTCATCCTTCTCCTGTTCATA** |

**Representative western blotting membranes from Supplementary figure 1:**

WB membranes probed with anti-rabbit phospho-AKT, anti-rabbit AKT, anti-rabbit phospho-AMPK and anti-rabbit AMPK. 1- Wt, 2- Cap, 3- T1D, 4- T1D+Cap, 5- 5LO^-/-^, 6- 5LO^-/-^+Cap, 7 -5LO^-/-^ T1D, and 8- 5LO^-/-^ T1D+Cap. Representative pAKT, AKT, pAMPK, and AMPK from the membranes with all samples 2.


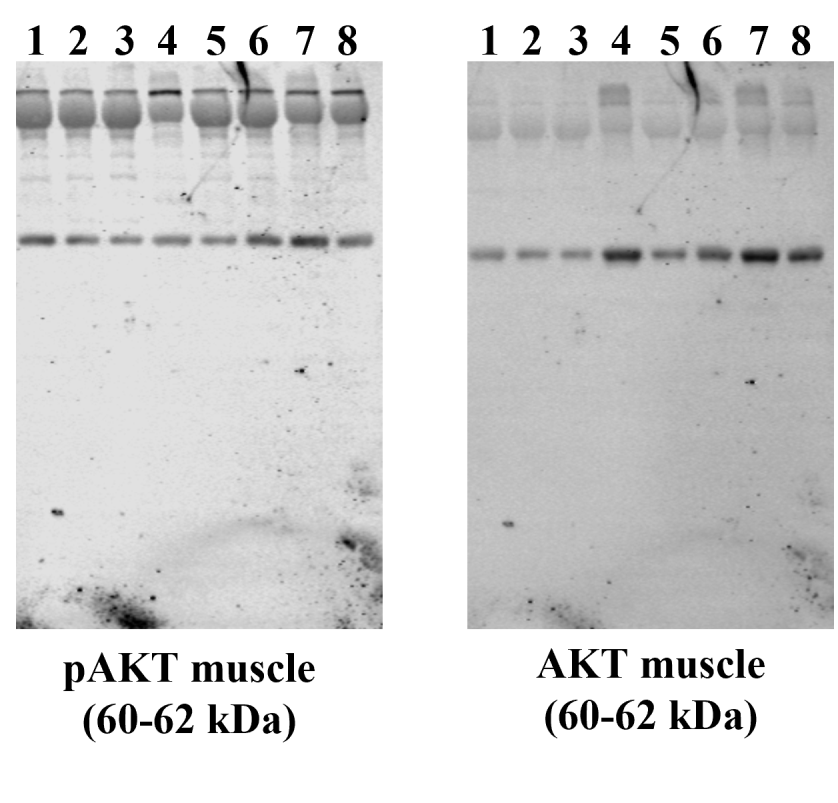


**~62kDa**

**~62kDa**


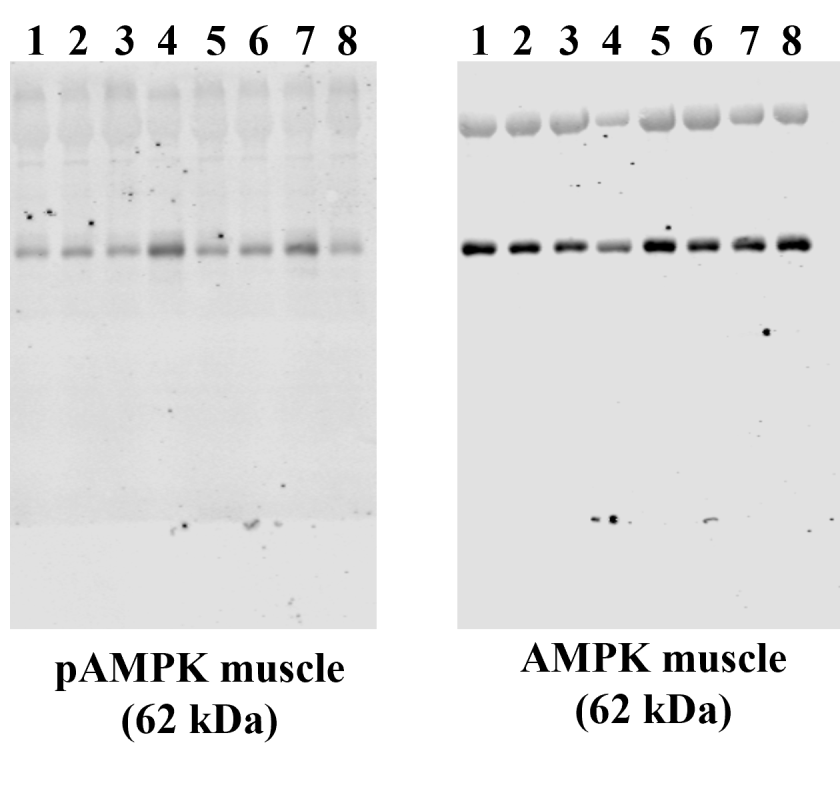


**~62kDa**

**~62kDa**

**Representative western blotting membranes from Supplementary figure 2:**

WB membranes probed with anti-rabbit phospho-AKT, anti-rabbit AKT, anti-rabbit phospho-AMPK and anti-rabbit AMPK. 1- Wt, 2- Cap, 3- T1D, 4- T1D+Cap, 5- 5LO^-/-^, 6- 5LO^-/-^+Cap, 7 -5LO^-/-^ T1D, and 8- 5LO^-/-^ T1D+Cap. Representative pAKT and AKT, from the membranes with all samples 3, and pAMK and AMPK, from the membranes with all samples 1 and 2, respectively.


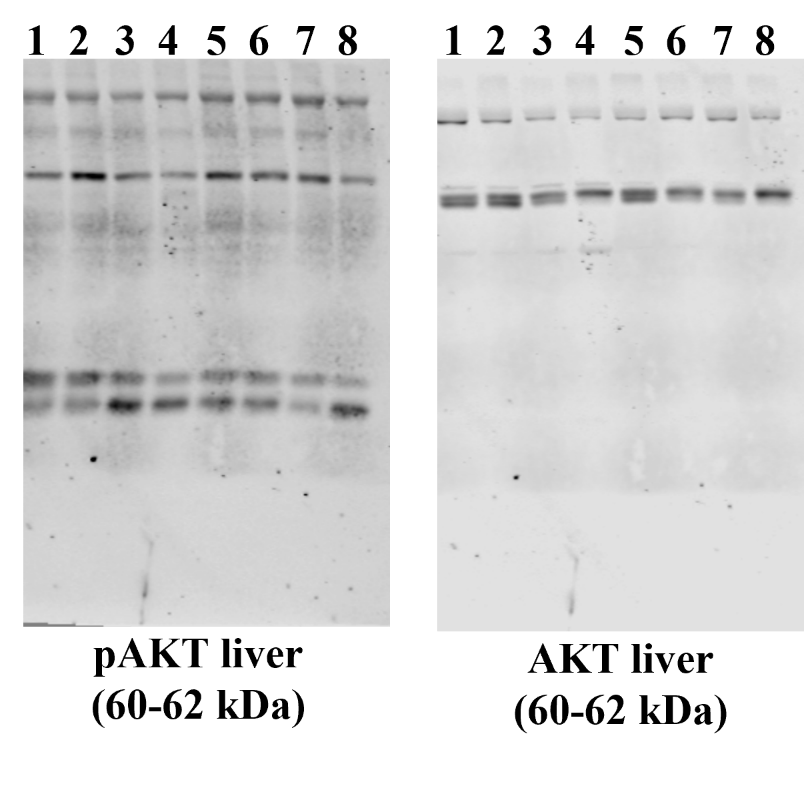


**~62kDa**

**~62kDa**


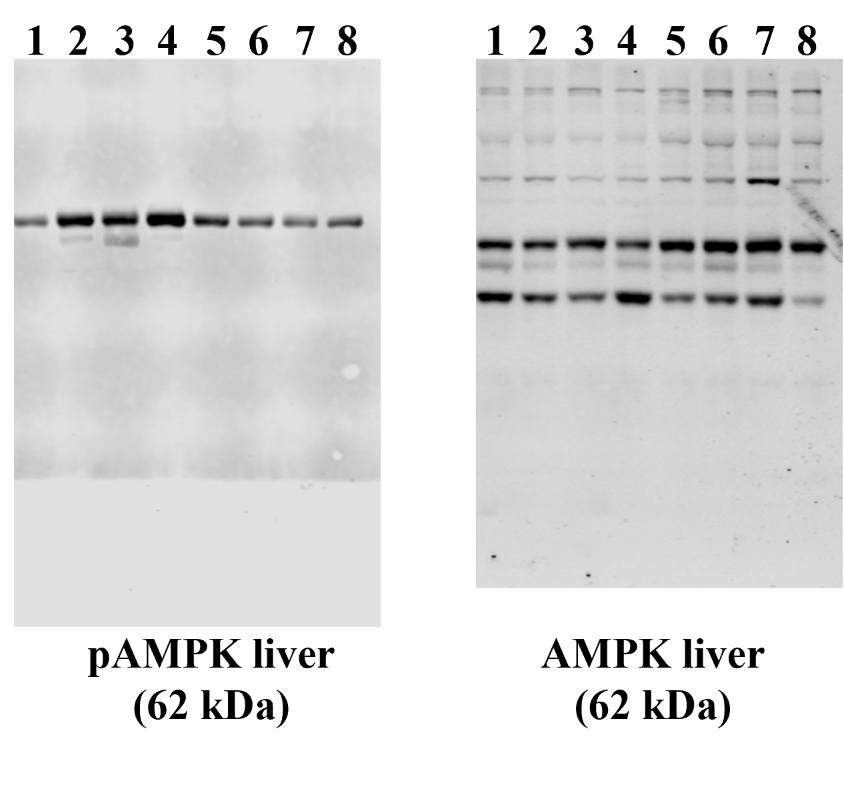


**~62kDa**

**~62kDa**

**Representative western blotting membranes from Supplementary figure 3:**

WB membranes probed with anti-rabbit phospho-mTOR, anti-rabbit mTOR, anti-rabbit LC3 and anti-rabbit TBP. 1- Wt, 2- Cap, 3- T1D, 4- T1D+Cap, 5- 5LO^-/-^, 6- 5LO^-/-^+Cap, 7 -5LO^-/-^ T1D, and 8- 5LO^-/-^ T1D+Cap. Representative pmTOR and mTOR, from the membranes with all samples 5, and LC3 and TBP, from the membranes with all samples 4.


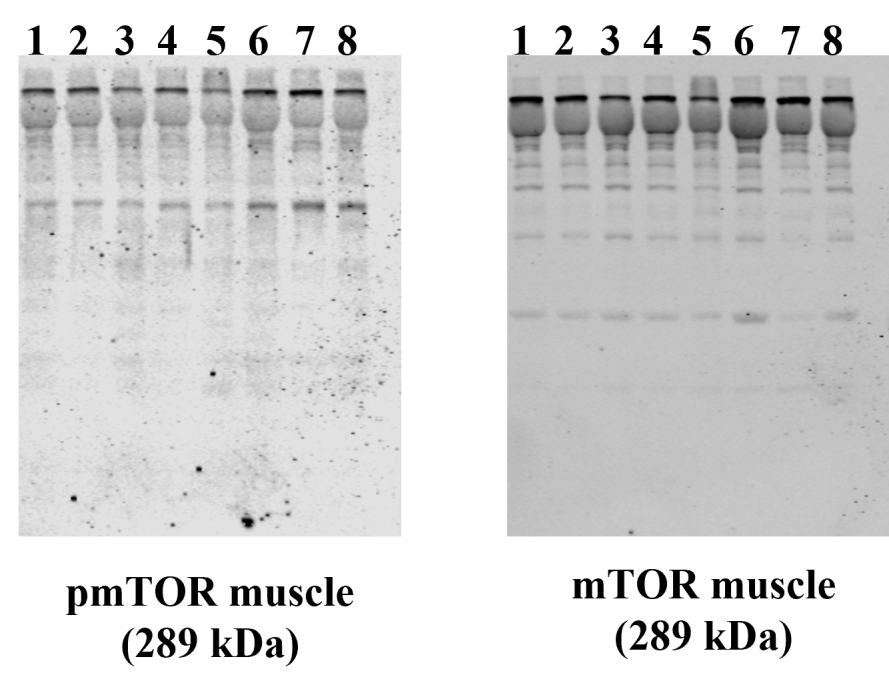


**~289kDa**

**~289kDa**


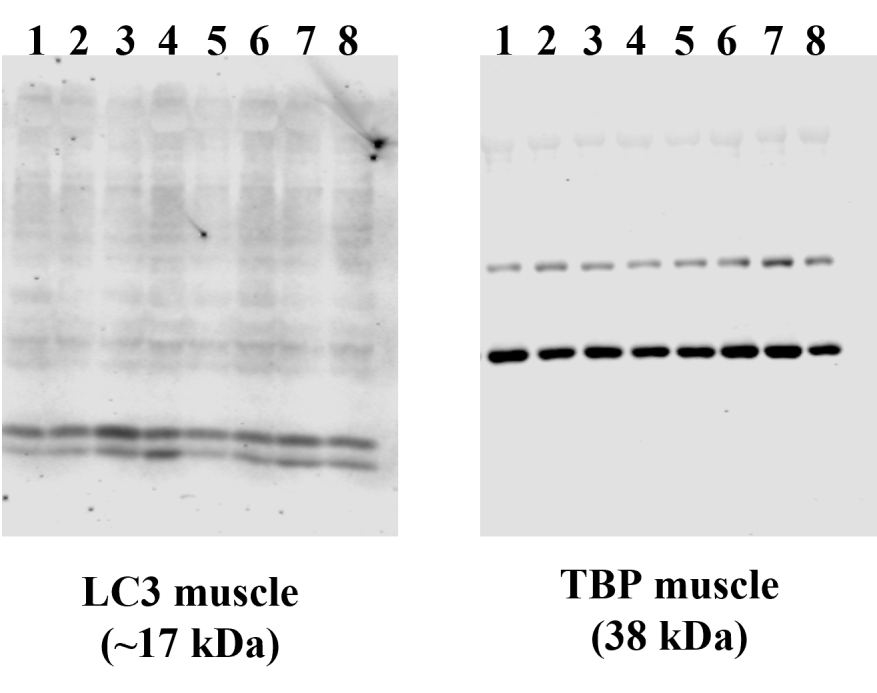


**~38kDa**

**~17kDa**

**Representative western blotting membranes from Supplementary figure 4:**

WB membranes probed with anti-rabbit phospho-mTOR, anti-rabbit mTOR, anti-rabbit LC3 and anti-rabbit TBP. 1- Wt, 2- Cap, 3- T1D, 4- T1D+Cap, 5- 5LO^-/-^, 6- 5LO^-/-^+Cap, 7 -5LO^-/-^ T1D, and 8- 5LO^-/-^ T1D+Cap. Representative pmTOR, mTOR, LC3, and TBP from the membranes with all samples 3.


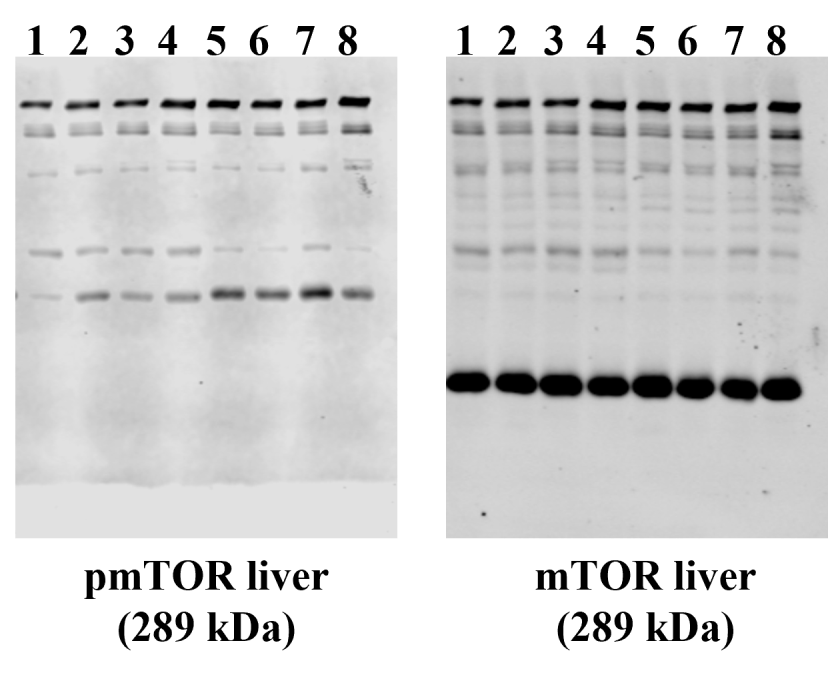


**~289kDa**

**~289kDa**


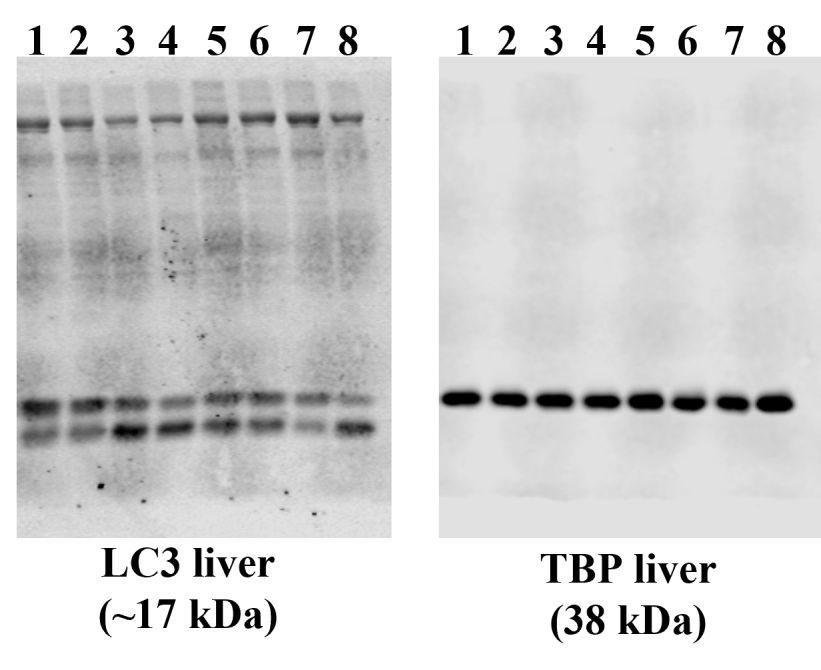


**~38kDa**

**~17kDa**

**All replicates used for the western blotting analysis for Supplementary figure 1:**

WB membranes probed with anti-rabbit phospho-AKT, anti-rabbit AKT, anti-rabbit phospho-AMPK and anti-rabbit AMPK. After the first 4 samples used for another study, the remaining 8, used for this one was pipetted as follows: 1- Wt, 2- Cap, 3- T1D, 4- T1D+Cap, 5- 5LO^-/-^, 6- 5LO^-/-^+Cap, 7 -5LO^-/-^ T1D, and 8- 5LO^-/-^ T1D+Cap.


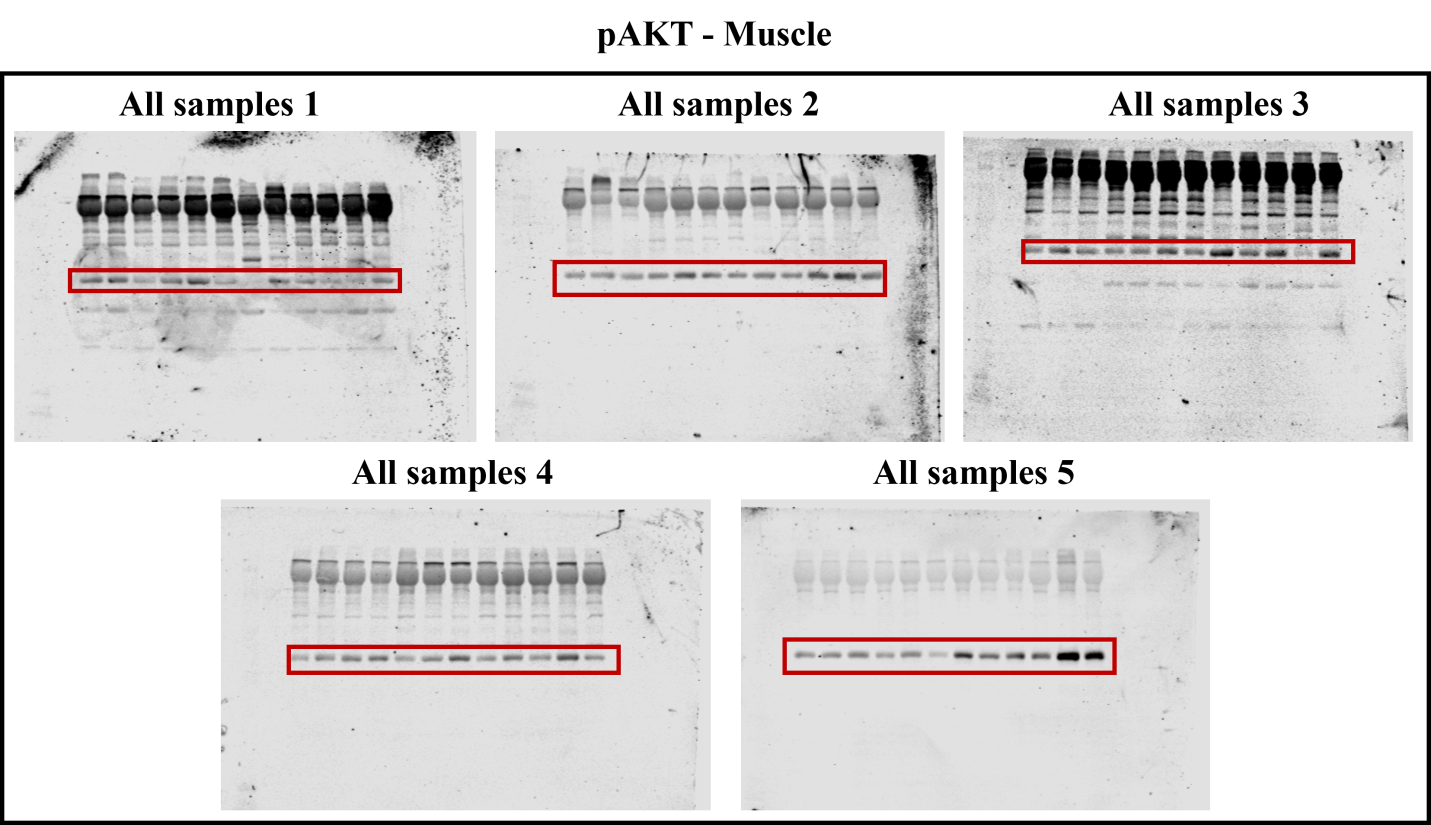


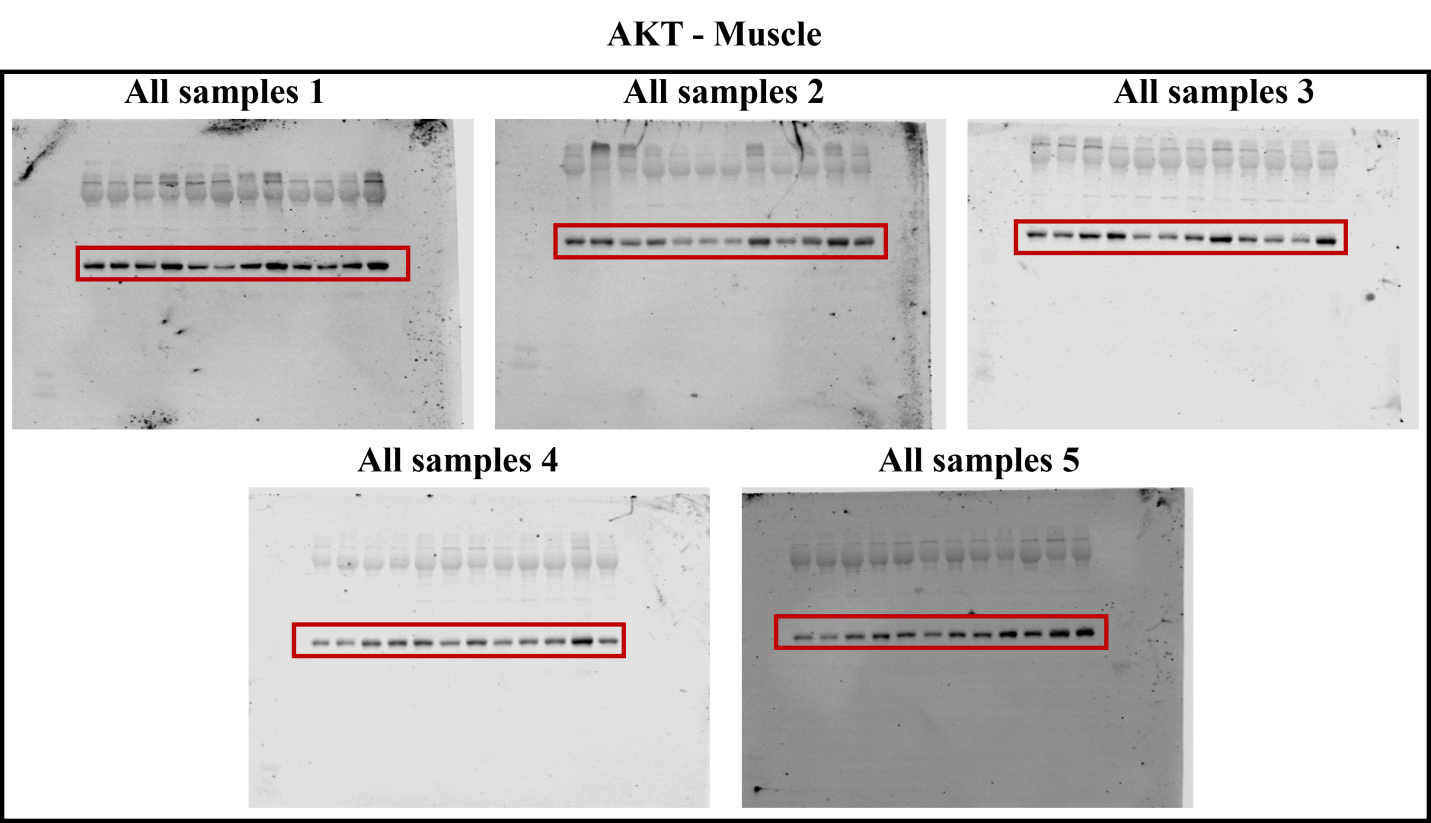


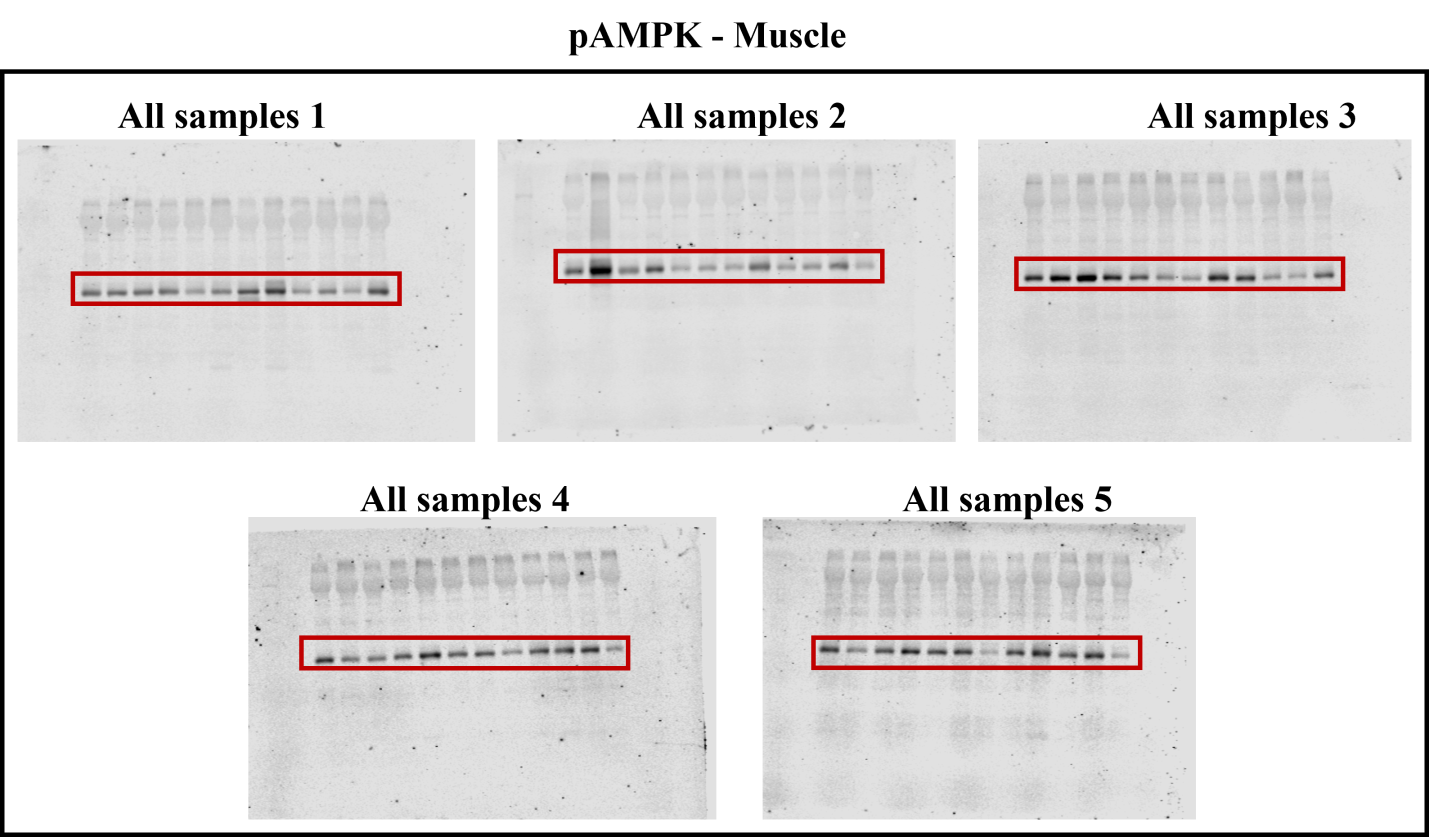


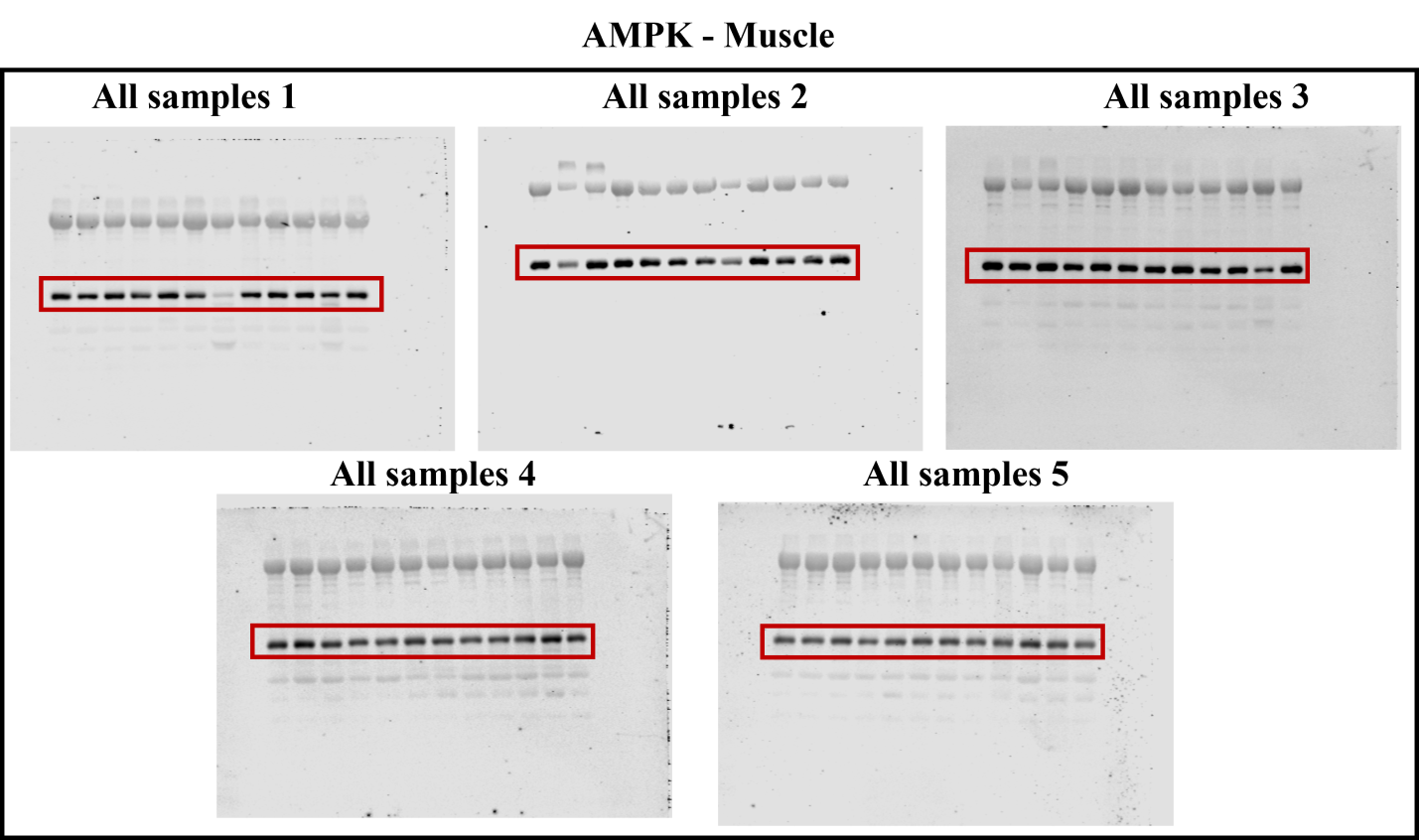


**All replicates used for the western blotting analysis for Supplementary figure 2:**

WB membranes probed with anti-rabbit phospho-AKT, anti-rabbit AKT, anti-rabbit phospho-AMPK and anti-rabbit AMPK. After the first 4 samples used for another study, the remaining 8, used for this one was pipetted as follows: 1- Wt, 2- Cap, 3- T1D, 4- T1D+Cap, 5- 5LO^-/-^, 6- 5LO^-/-^+Cap, 7 -5LO^-/-^ T1D, and 8- 5LO^-/-^ T1D+Cap.


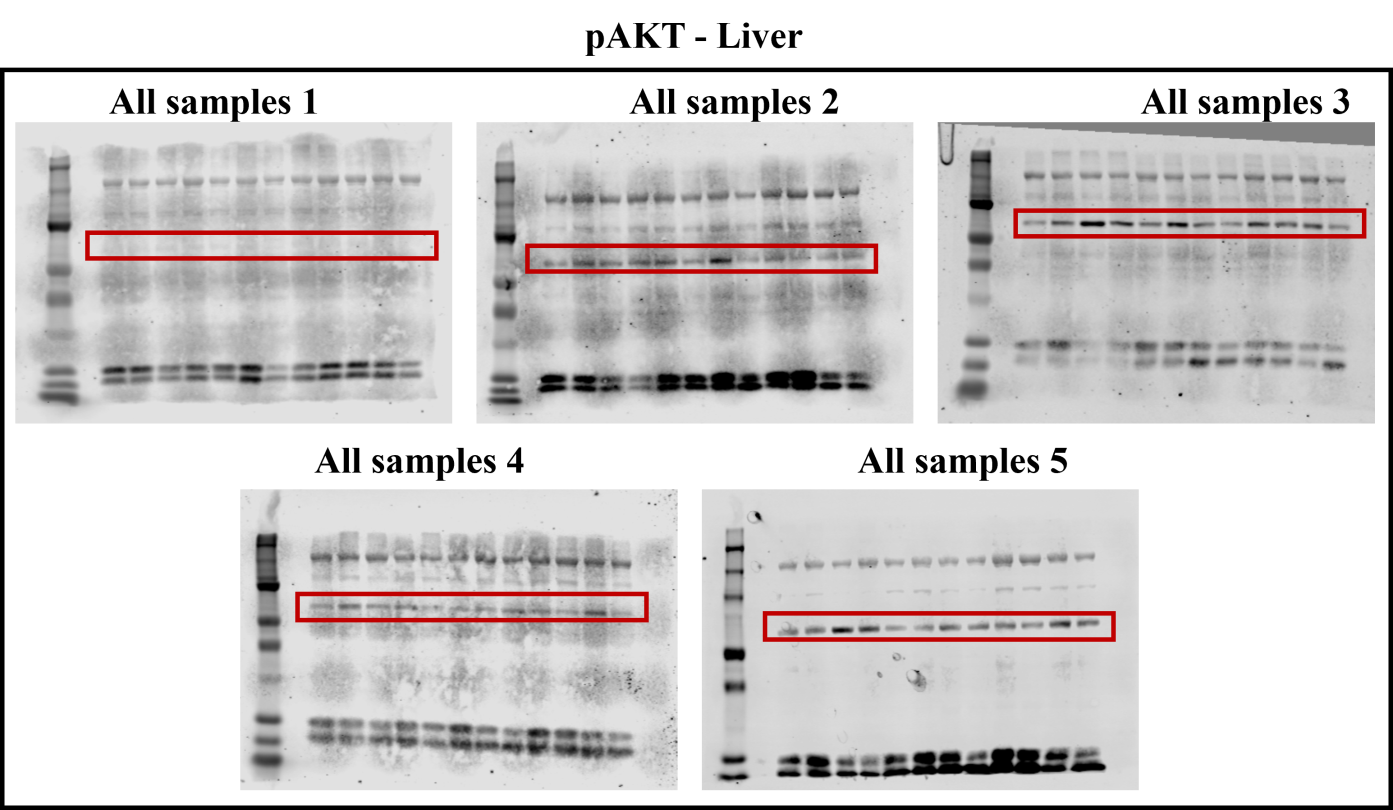


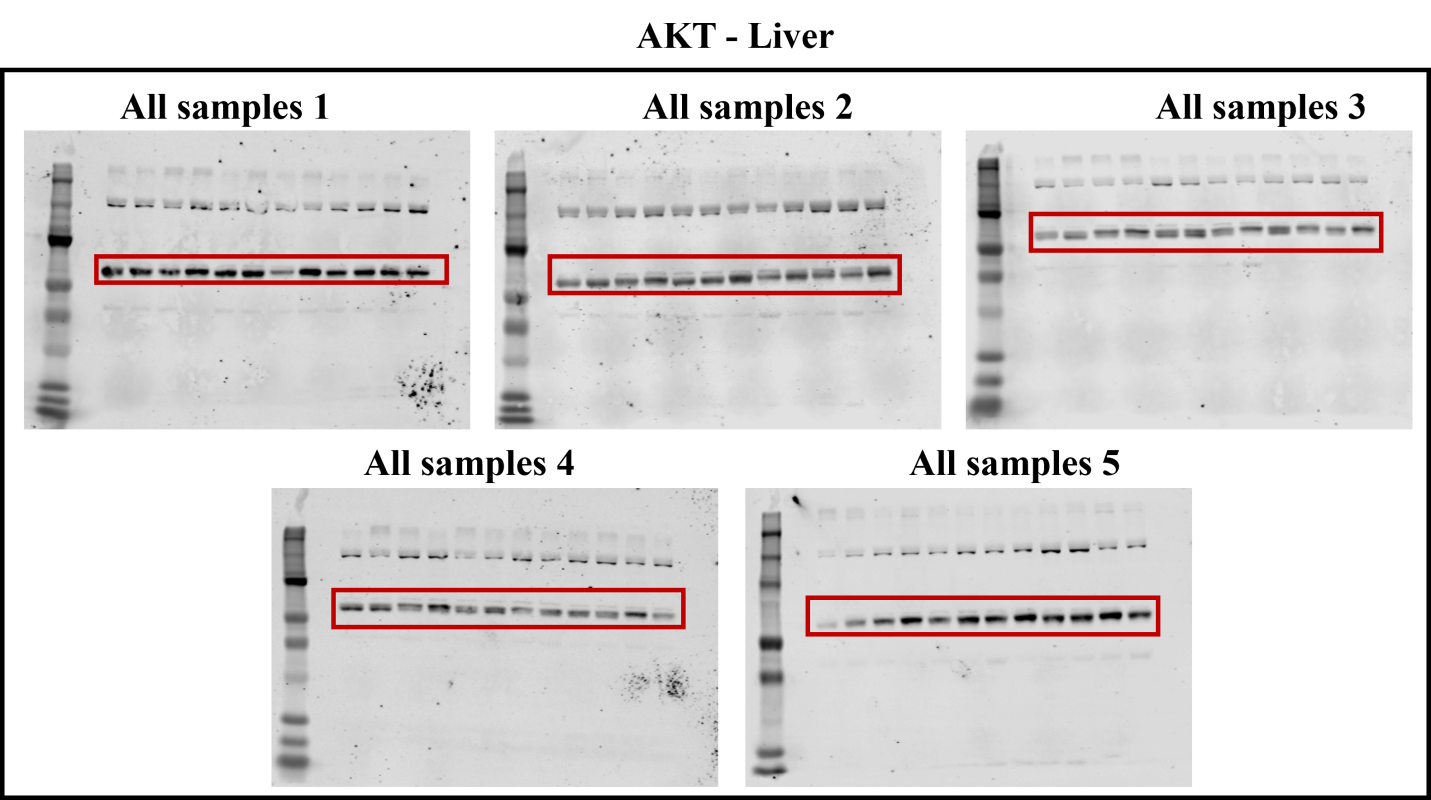


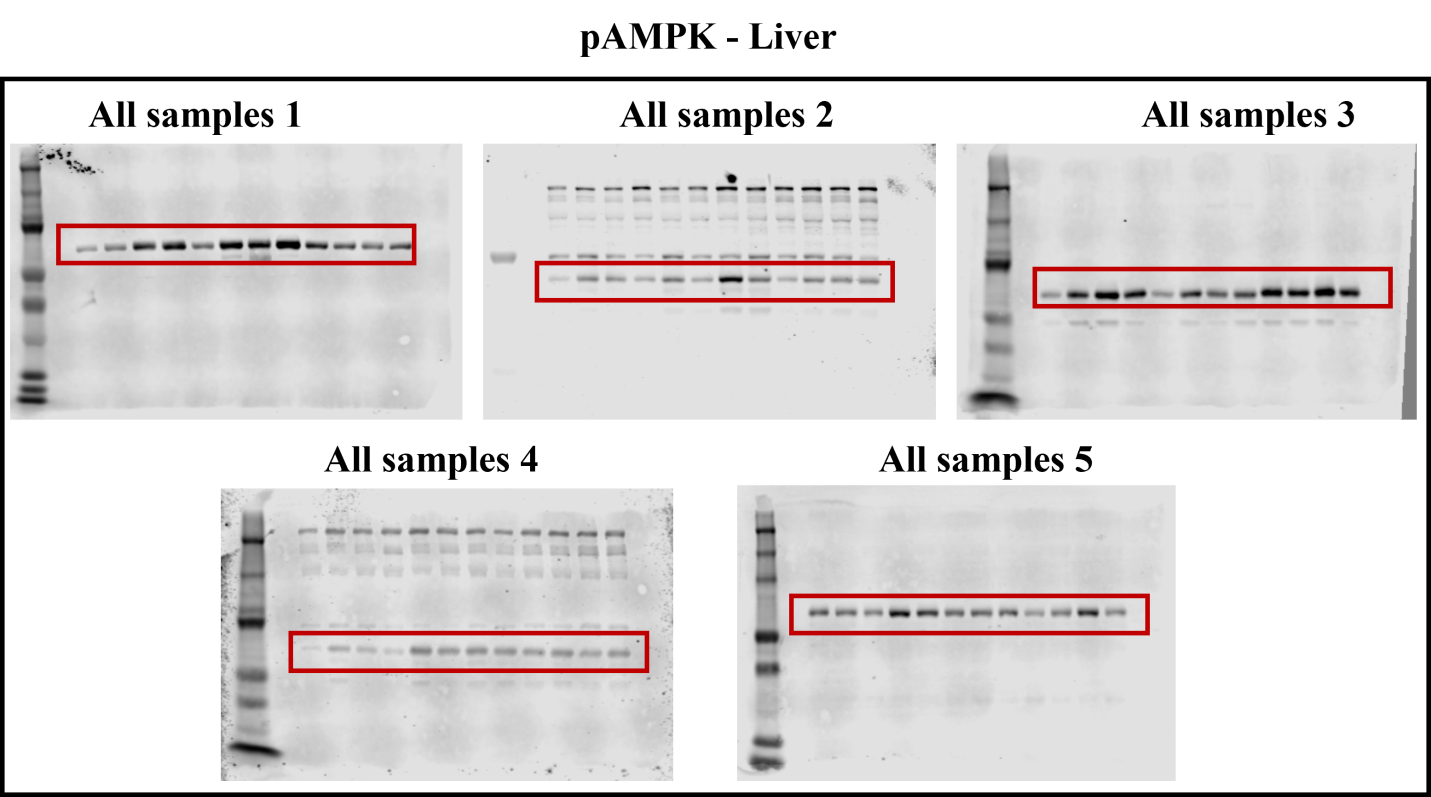


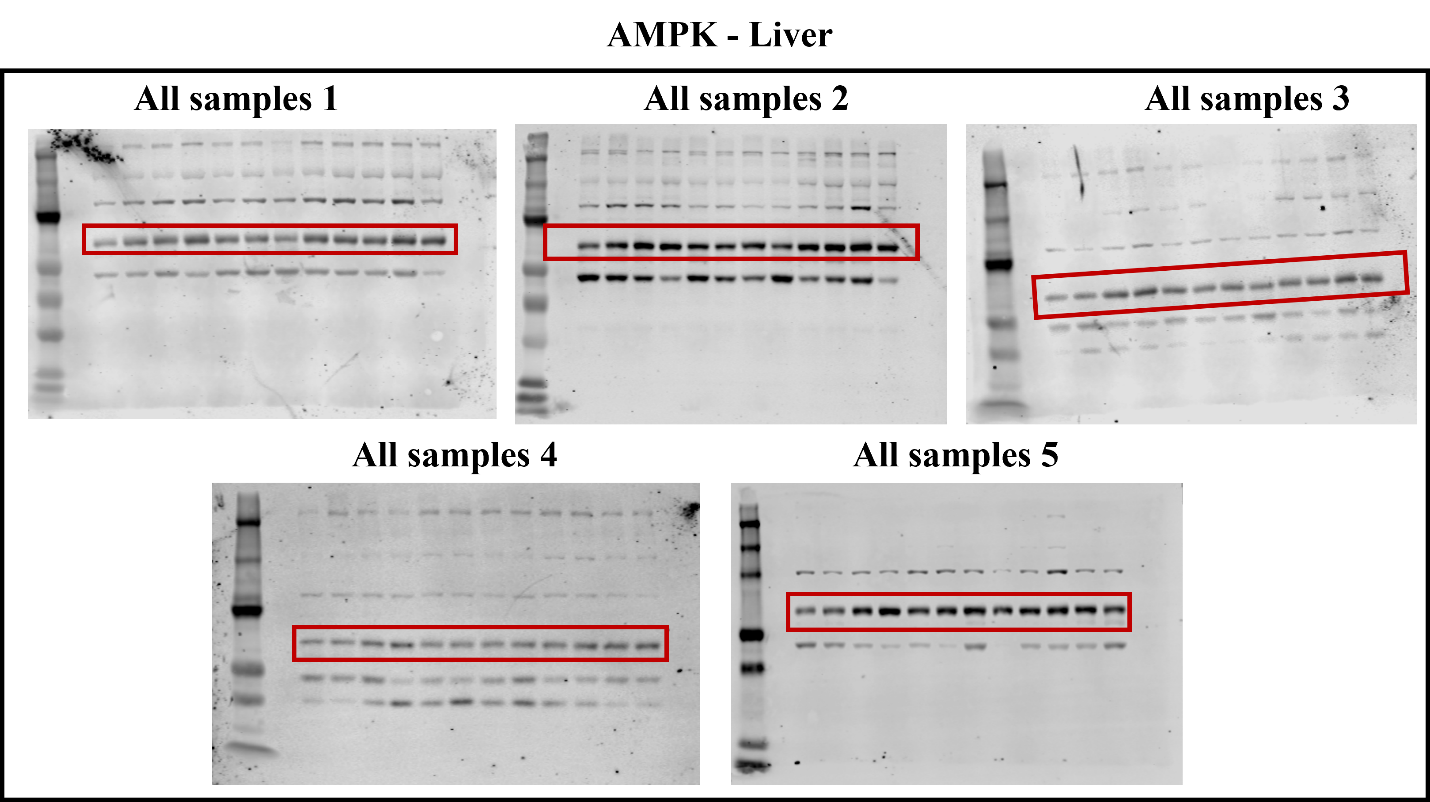


**Representative western blotting membranes from Supplementary figure 3:**

WB membranes probed with anti-rabbit phospho-mTOR, anti-rabbit mTOR, anti-rabbit LC3 and anti-rabbit TBP. After the first 4 samples used for another study, the remaining 8, used for this one was pipetted as follows: 1- Wt, 2- Cap, 3- T1D, 4- T1D+Cap, 5- 5LO^-/-^, 6- 5LO^-/-^+Cap, 7 -5LO^-/-^ T1D, and 8- 5LO^-/-^ T1D+Cap.


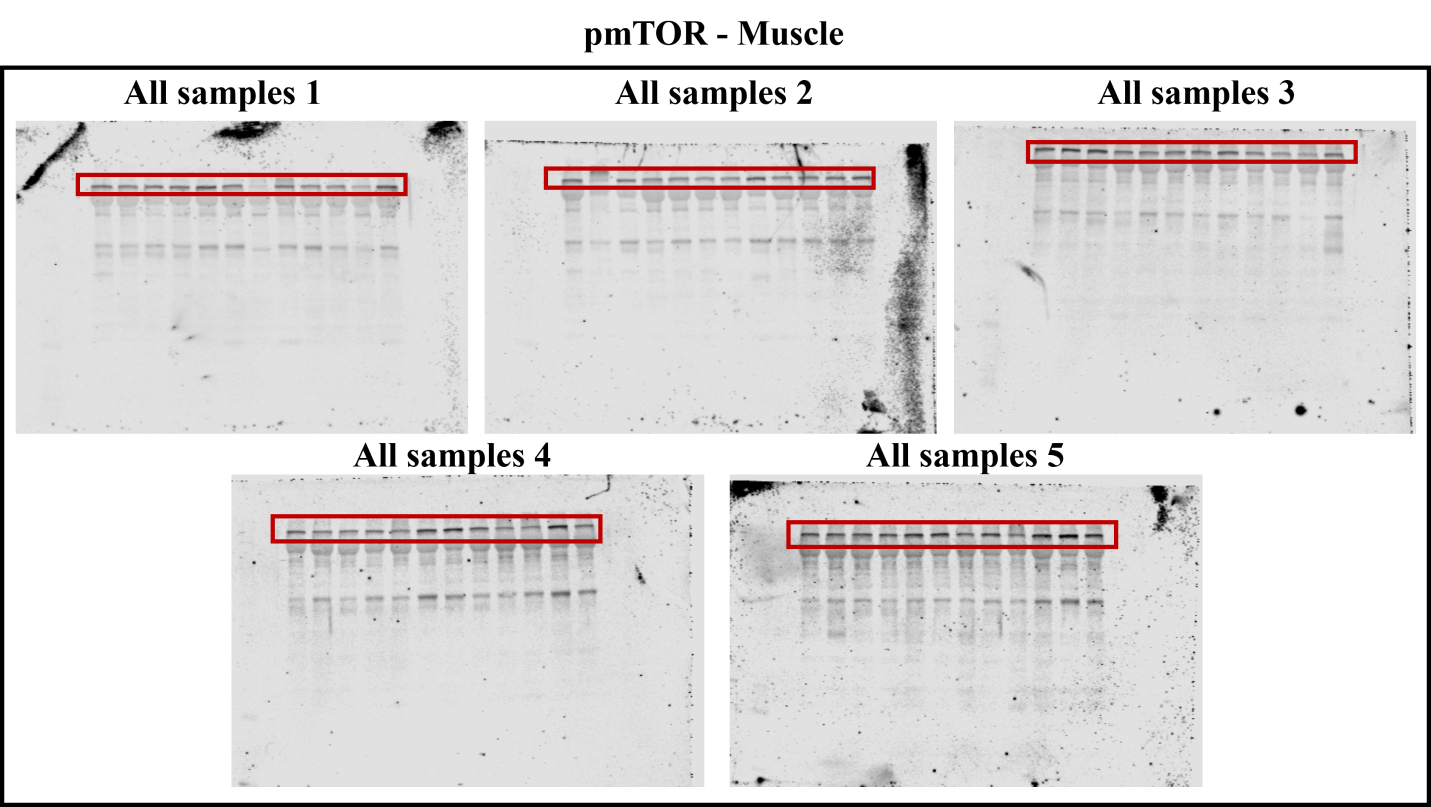


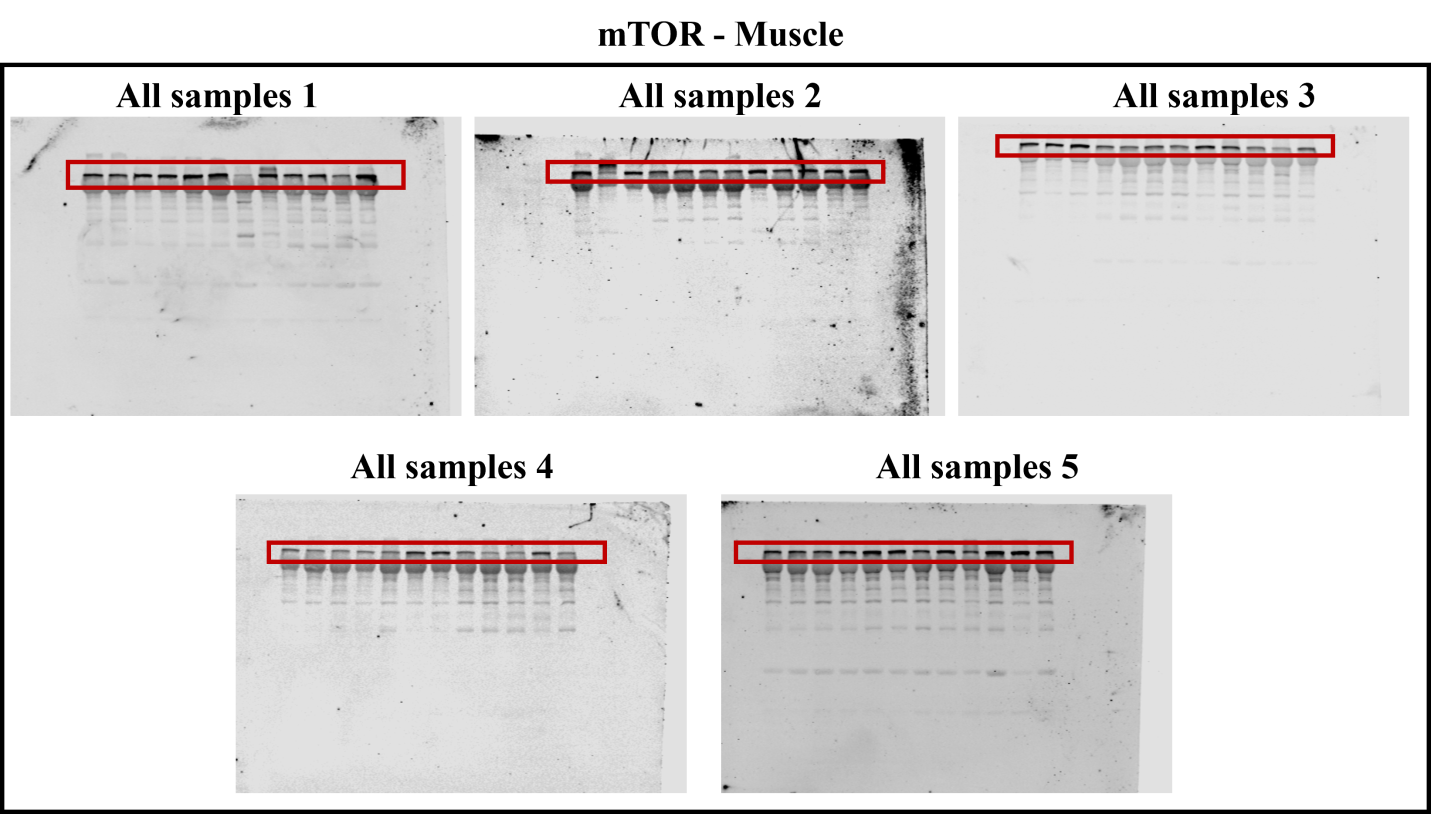


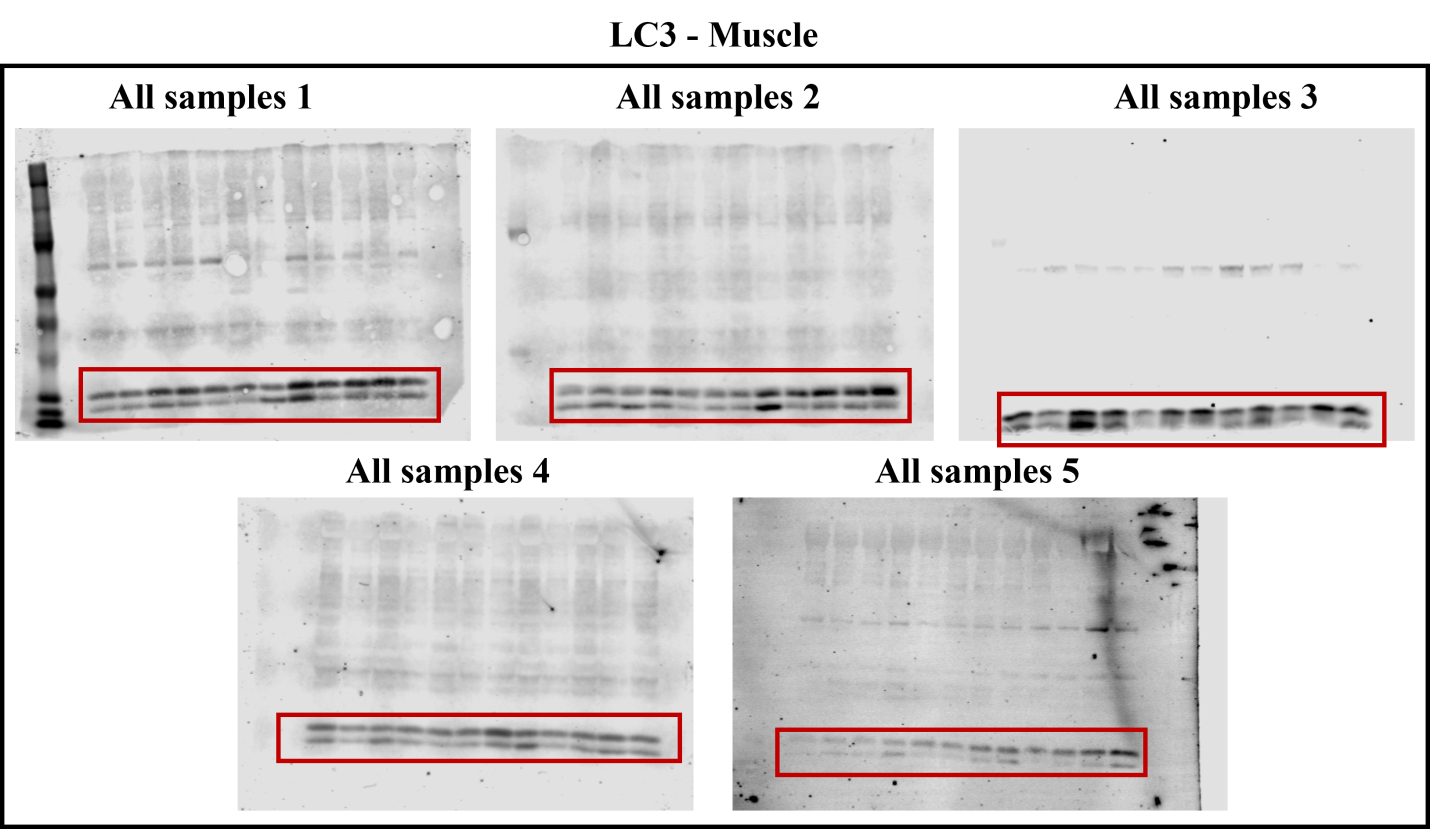


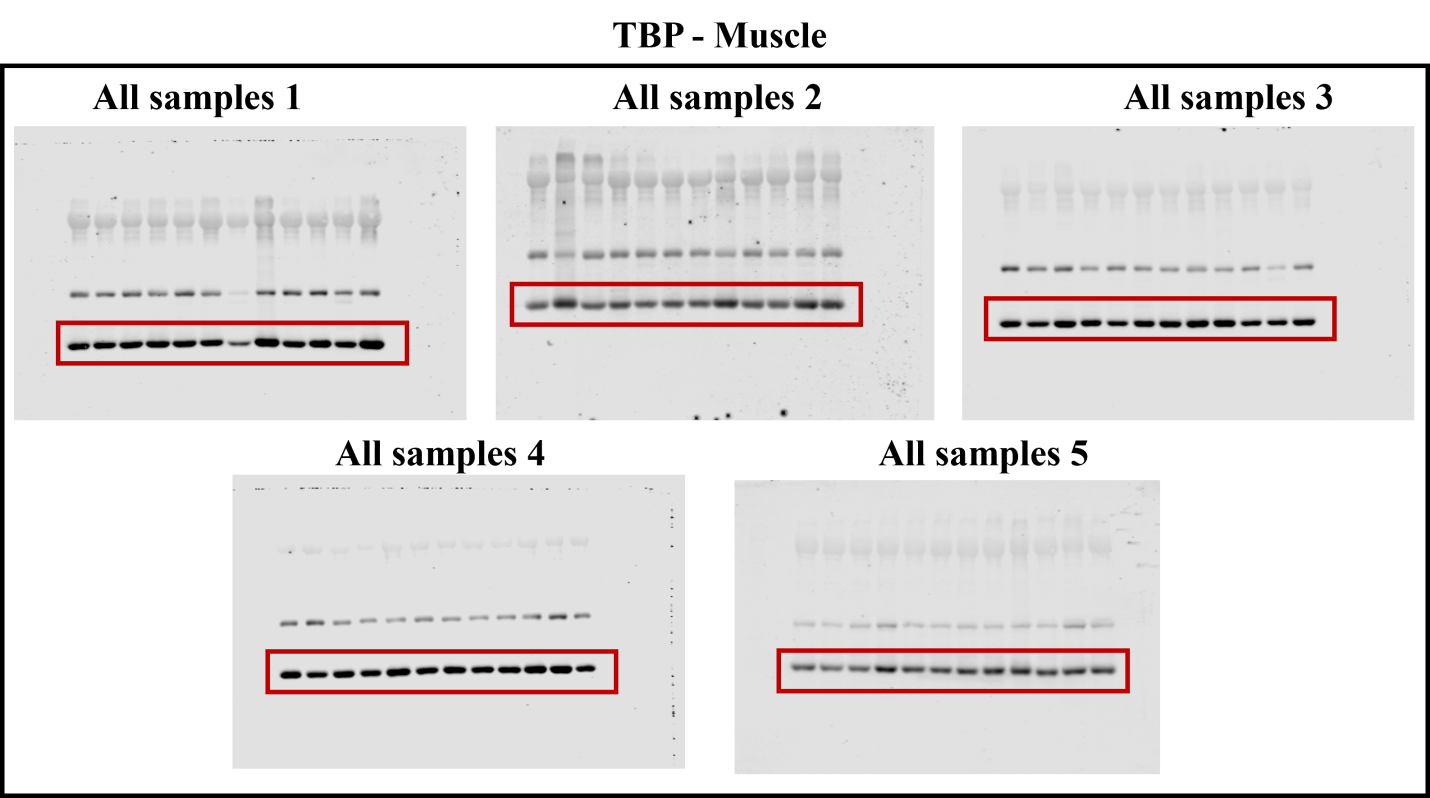


**Representative western blotting membranes from Supplementary figure 4:**

WB membranes probed with anti-rabbit phospho-mTOR, anti-rabbit mTOR, anti-rabbit LC3 and anti-rabbit TBP. After the first 4 samples used for another study, the remaining 8, used for this one was pipetted as follows: 1- Wt, 2- Cap, 3- T1D, 4- T1D+Cap, 5- 5LO^-/-^, 6- 5LO^-/-^+Cap, 7 -5LO^-/-^ T1D, and 8- 5LO^-/-^ T1D+Cap.


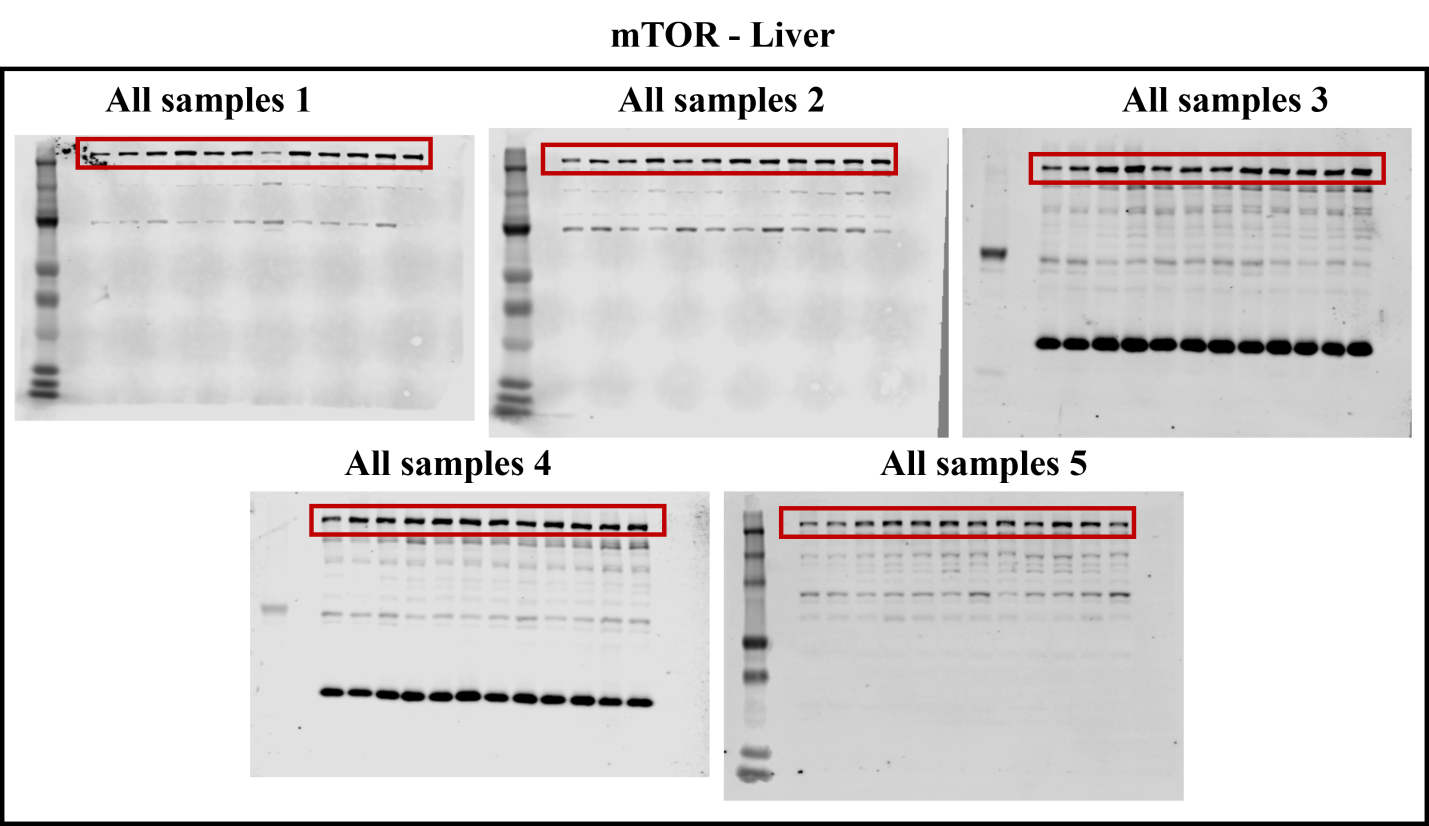

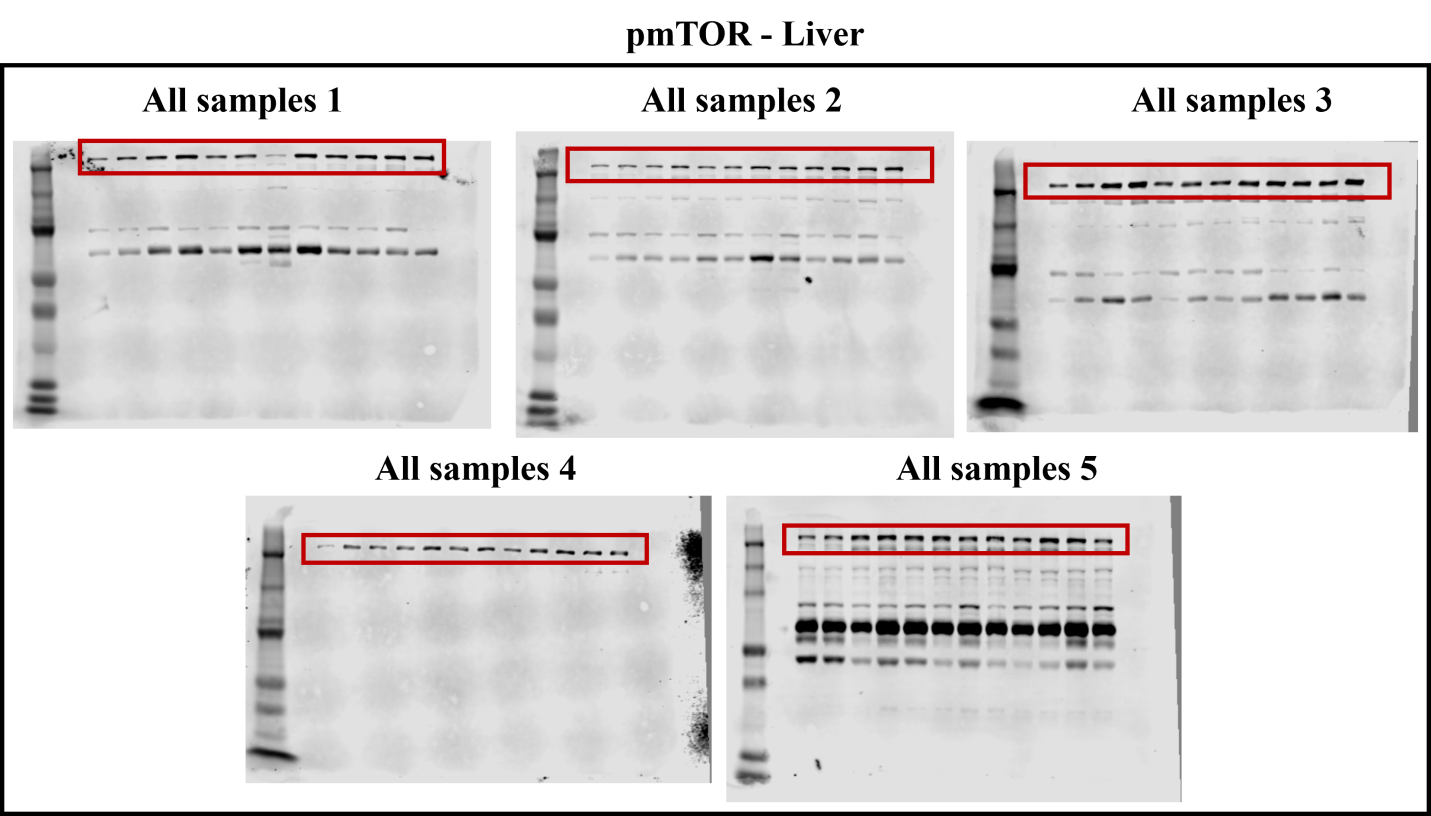


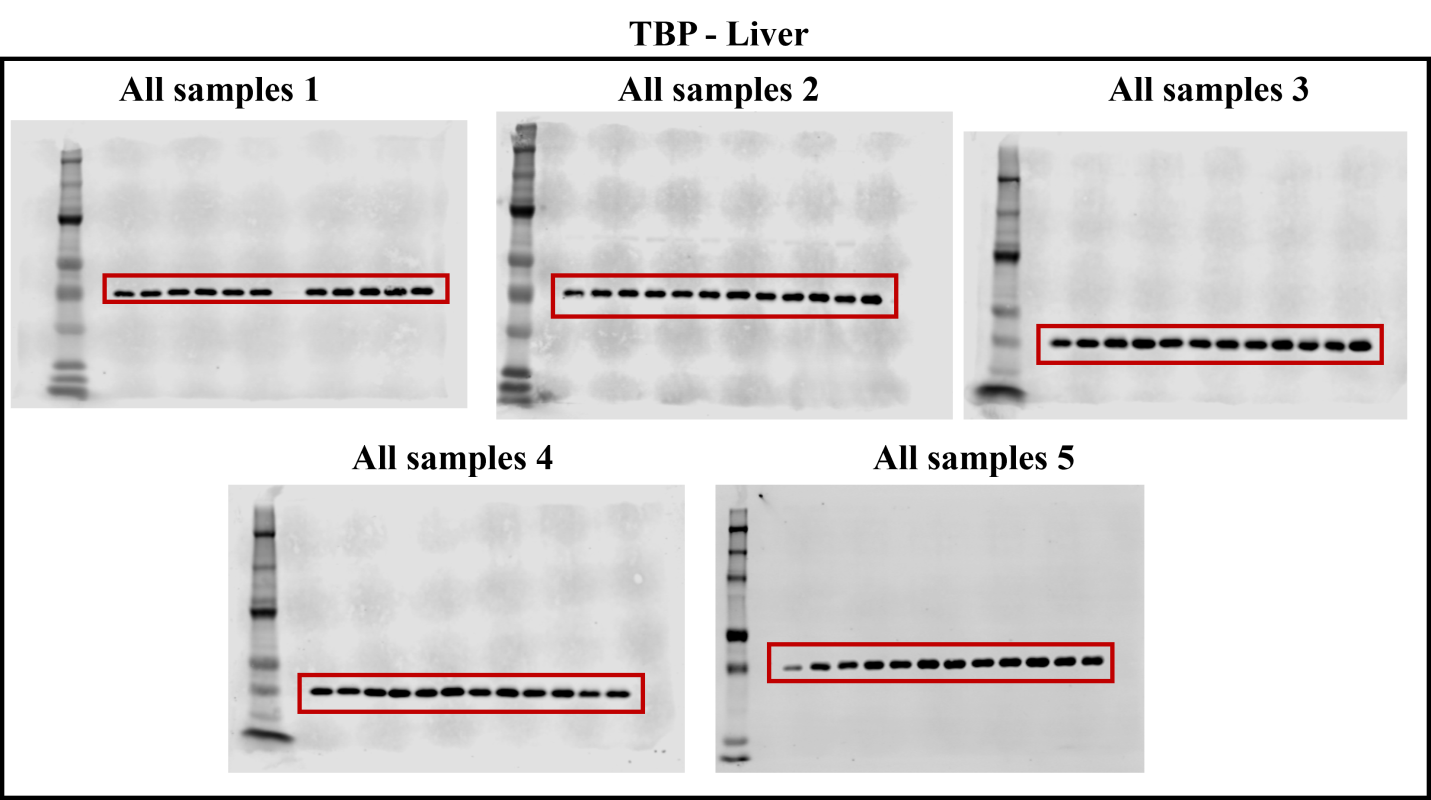

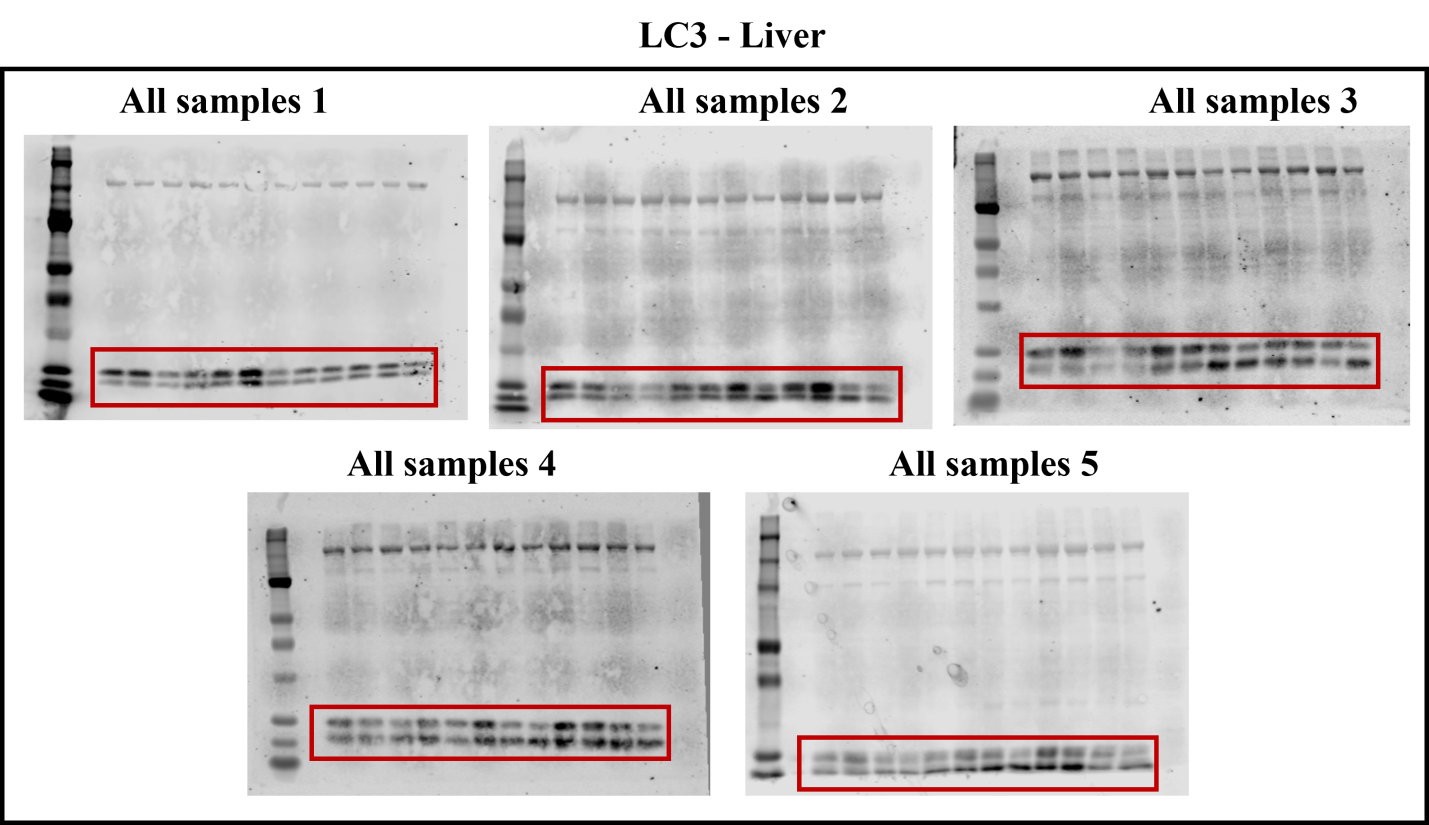

Supplement: Supplementary file 1 — Supplementary Information. [file 41598_2023_49449_MOESM1_ESM.docx]
